# Identification and expression analysis of the apple (*Malus* × *domestica*) basic helix-loop-helix transcription factor family

**DOI:** 10.1038/s41598-017-00040-y

**Published:** 2017-02-09

**Authors:** Jinhua Yang, Min Gao, Li Huang, Yaqiong Wang, Steve van Nocker, Ran Wan, Chunlei Guo, Xiping Wang, Hua Gao

**Affiliations:** 1 State Key Laboratory of Crop Stress Biology in Arid Areas, College of Horticulture, Northwest A&F University, Yangling, China; 2 Key Laboratory of Horticultural Plant Biology and Germplasm Innovation in Northwest China, Ministry of Agriculture, Northwest A&F University, Yangling, China; 3 Department of Biological Sciences, University of Maryland, Baltimore County, 1000 Hilltop Circle, Baltimore, MD 21250 USA; 4 Department of Horticulture, Michigan State University, East Lansing, MI 48824 USA

## Abstract

Basic helix-loop-helix (bHLH) proteins, which are characterized by a conserved bHLH domain, comprise one of the largest families of transcription factors in both plants and animals, and have been shown to have a wide range of biological functions. However, there have been very few studies of bHLH proteins from perennial tree species. We describe here the identification and characterization of 175 bHLH transcription factors from apple (*Malus* × *domestica*). Phylogenetic analysis of apple bHLH (*MdbHLH*) genes and their *Arabidopsis thaliana* (Arabidopsis) orthologs indicated that they can be classified into 23 subgroups. Moreover, integrated synteny analysis suggested that the large-scale expansion of the bHLH transcription factor family occurred before the divergence of apple and Arabidopsis. An analysis of the exon/intron structure and protein domains was conducted to suggest their functional roles. Finally, we observed that *MdbHLH* subgroup III and IV genes displayed diverse expression profiles in various organs, as well as in response to abiotic stresses and various hormone treatments. Taken together, these data provide new information regarding the composition and diversity of the apple bHLH transcription factor family that will provide a platform for future targeted functional characterization.

## Introduction

Numerous plant-specific transcription factors (TFs) have been shown to play important roles regulating the development of plant-specific organs and adaptations to terrestrial environments^1^. The bHLH proteins constitute one of the largest TF families and share a conserved domain of approximately 60 amino acids, including a 15-amino acid basic region and a HLH region that contains two amphipathic α-helices with a linking loop that varies in length^2^. The basic region is involved in binding to specific DNA sequences, while dimerization with other HLH-containing proteins occurs through the HLH region, and is a prerequisite for DNA binding^2^.

Since the bHLH domain was initially defined^2^, a variety of bHLH subgroups have been identified based on the frequencies of 19 conserved amino acids in the bHLH consensus motif^2^. Structural analyses of animal bHLH proteins indicated that they comprise only six groups (designated A-F)^3^, while phylogenetic analyses have shown that plant bHLH proteins comprise 26 subgroups, twenty of which are present in the common ancestors of extant mosses and vascular plants, and six additional subgroups that evolved among the vascular plants^4^.

In animals, bHLH proteins are involved in regulating responses to environmental signals, controlling cell cycle and circadian rhythms, and modulating a range of developmental processes, such as neurogenesis, myogenesis, sex and cell lineage determination, proliferation, and differentiation^3,5^. In addition, functions in processes such as chromosome segregation, general transcriptional enhancement, and metabolism regulation have been demonstrated in unicellular eukaryotes, such as budding yeast^6^. In plants, the founding member of the bHLH superfamily is the maize (*Zea mays*) R gene, which plays a key role in anthocyanin biosynthesis^7^. Subsequently, many more functions for plant bHLH genes have been identified, including the regulation of light signaling^8,9^; hormone signal transduction^10^; responses to wounding, drought, salt and oxidative stresses^11^ and low temperatures^12^; iron deficiency^13^; symbiotic ammonium transport^14^ and flavonoid synthesis^15^. In addition, plant bHLH TFs also influence the development of shoot branches^16^, fruits and flowers^17,18^, microspores^19^, trichomes^20,21^, stomata^22^ and roots^23^.

Despite studies of the plant bHLH TFs from different plant species, only a few have previously been characterized in perennial tree species^24^. In this study, we explored the evolution and structure of the bHLH TF family in apple (*Malus* × *domestica* Borkh) through phylogenetic analysis and integrated synteny analysis with orthologs from the model plant *Arabidopsis thaliana* (Arabidopsis), combined with exon/intron structural analysis. These analyses provide evidence that the bHLH domain is highly conserved and that the bHLH TFs from these two species share a common ancestor. In addition, we evaluated the expression profiles of *MdbHLH* genes in ten different plant structures, and measured their transcript abundance in response to different phytohormone treatments and following exposure to high-salt stress. These results revealed that the bHLH TFs exhibit a wide range of expression patterns, indicating functional diversity. This study represents an important step in elucidating the biological and molecular functions of apple bHLH TFs, as well their evolutionary diversification.

## Results

### Genome-wide identification of apple bHLH TF protein encoding genes

To identify apple bHLH protein encoding genes, we searched the predicted apple proteome using an HMM algorithm (HMMER) with the bHLH conserved domain (PF00010) and the definition of bHLH proteins reported by Atchley *et al.* ^2^. After re-checking the structural integrity of the conserved domains using SMART software (http://smart.emblheidelbergde/) and removing any redundant proteins, a total of 175 bHLH proteins were defined. Based on a multiple sequence alignment (Supplementary Fig. S1), the proteins were named sequentially as *MdbHLH001* to *MdbHLH175*, based on the equivalent classification in Arabidopsis and rice (*Oryza sativa*)^4^. At least one expressed sequence tag (EST) was identified for each of 82 *MdbHLH* genes from the NCBI apple ETS database (Supplementary Table S1). Interestingly, *MdbHLH142* had two HLH domains, with Expect (E)-values of 3.65E-17 and 2.77E-08 and sequence identity of 33%, a phenomenon reported in other species, including *Caenorhabditis elegans* and rice^25,26^. However, the biological function of this type of bHLH protein remains to be determined.

Detailed information regarding each member of the apple bHLH family, including gene locus numbers, accession numbers for the full-length sequences deposited at NCBI, chromosomal location, the length of protein sequences, open reading frames, and phylogeny relationship to Arabidopsis bHLH proteins, is listed in Supplementary Table S2. The annotation process revealed a substantial range in the lengths of the predicted proteins, from 92 to 1,397 amino acids, suggesting that the apple bHLH family may have a long evolutionary history and contribute to numerous biological processes.

**Figure 1:**
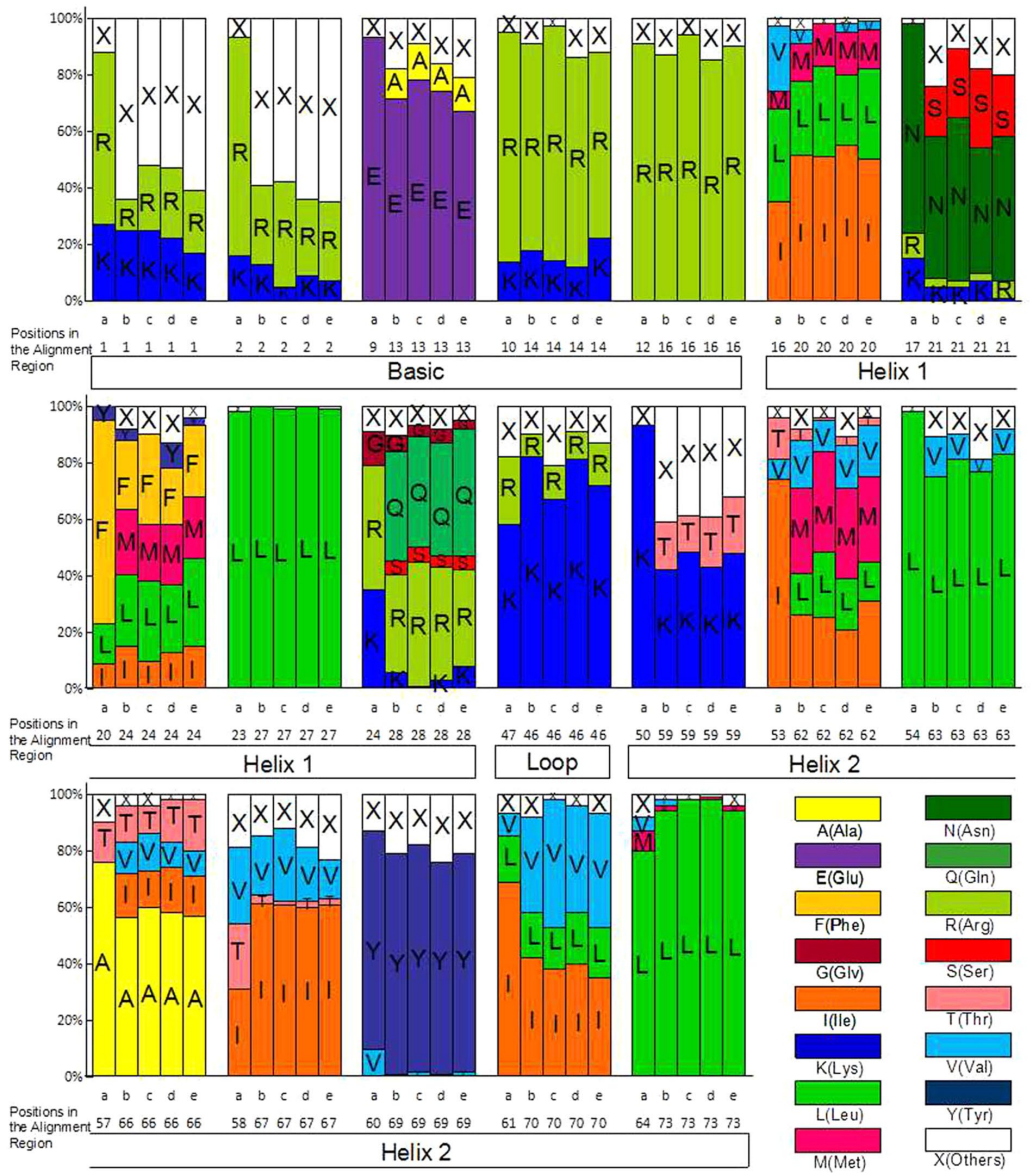
Amino acid distribution in the bHLH consensus motif. In columns labeled (a), percentage refers to the 392 animal bHLH proteins identified by Atchely *et al.* ^2^. In columns labeled (b,d and e), percentage refers to the 158 *AtbHLH* genes, the 183 *PtbHLH* genes and the 173 *OsbHLH* genes, respectively, as reported by Carretero-Paulet *et al.* ^28^. In columns labeled (c), percentage refers to the 175 *MdbHLH* genes identified in this current study. The numbers below (a,b,c,d and e) refer to the positions of the residues in the alignments of the studies.

Sequence conservation within the MdbHLH motifs was evaluated in the context of known bHLH proteins from animals, Arabidopsis, rice (*Oryza sativa*) and poplar (*Populus trichocarpa*)^2,27,28^. bHLH proteins are known to share 19 residues that are homologous between family members: five in the basic region, five in the first helix region, one in the loop region and eight in the second helix region. Typically, a protein is categorized as a bHLH protein if it contains 11 or more of these residues^2,27^, or nine or ten for group C and D bHLH proteins, because these groups do not have typical basic regions^29^, but are capable of binding to other bHLH proteins^30^. The conservation of 11 conserved residues (Glu-13, Arg-14, Arg-16, Asn-21, Leu-27, Lys-46, Leu-63, Ala-66, Ile-67, Tyr-69, and Leu-73 in our alignment) of the 19 conserved residues is shown in Fig. 1, demonstrating that the bHLH motifs are much more closely related among plant bHLH proteins than between bHLH proteins from plants and animals. The Leu-27 residue is highly conserved among bHLH proteins from all the examined organisms, suggesting that it may play an important role in the dimerization, which commonly occurs between bHLH proteins^27,28^.

### Phylogenetic analysis of apple and Arabidopsis bHLH genes

**Figure 2:**
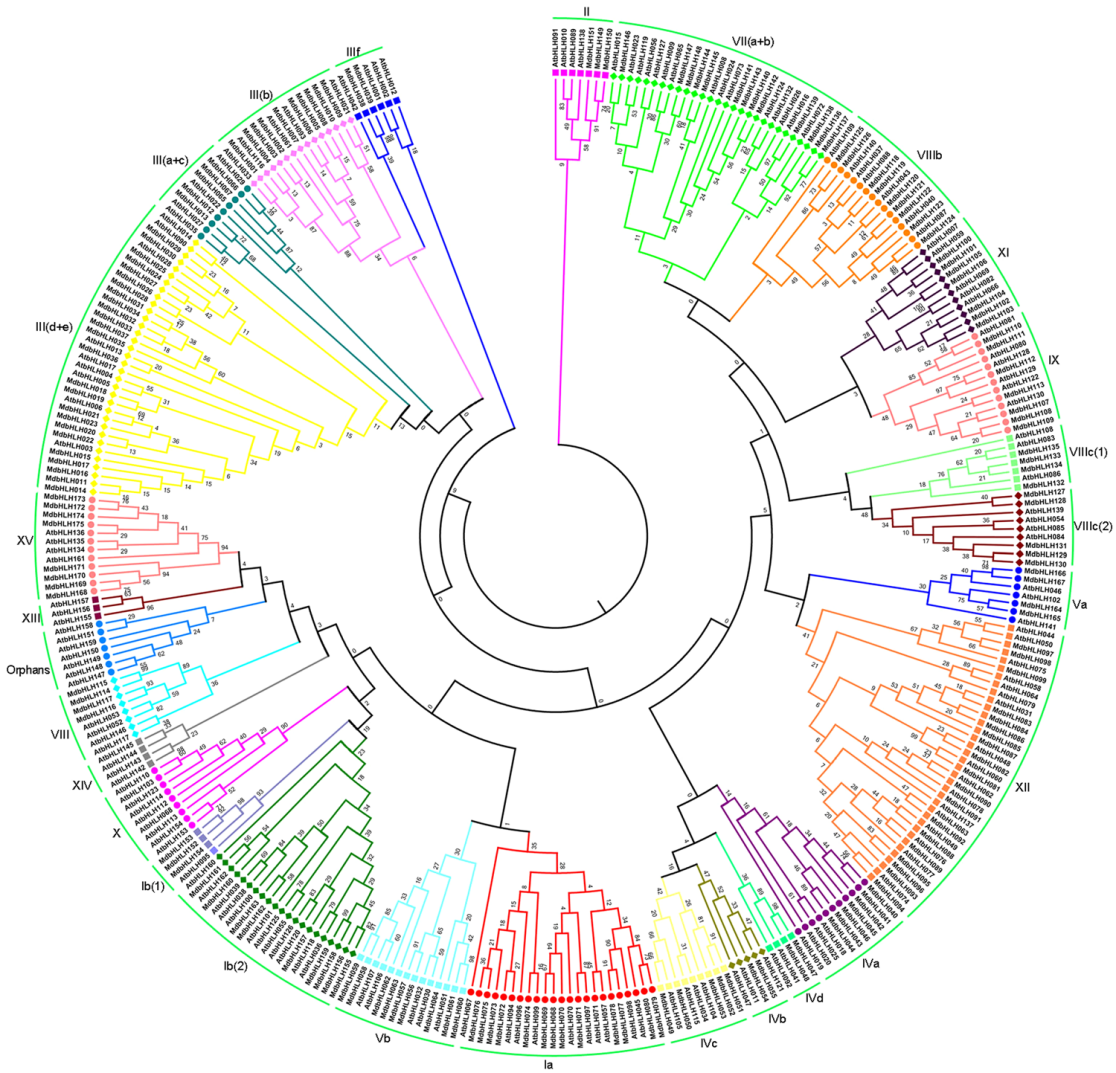
Phylogenetic tree based on apple and Arabidopsis bHLH transcription factors. The phylogenetic tree was generated with CLUSTALW and using the neighbor-joining method. The phylogenetic tree was inferred using MEGA 5.0 software. Reliability of the predicted tree was tested using bootstrapping with 1000 replicates. Numbers at the nodes indicate how often the group to the right appeared among bootstrap replicates. Branch lines of subtrees are colored indicating different bHLH subgroups. Apple and Arabidopsis *bHLH* genes in the same subgroup are denoted by squares (□), circles (○), triangles (Δ) and diamonds (◇) in various colors respectively.

To understand the evolutionary relationship of the bHLH TFs between species, a phylogenetic tree was constructed using the 175 apple bHLH sequences and 158 sequences from Arabidopsis (Fig. 2). The apple bHLH genes were distributed among 23 of the 26 total clades (subgroups), whereas some bHLH proteins did not align within any particular subgroup, and were thus termed ‘orphans’^4^.

To provide further insight into the phylogenetic relationships, we assessed the number of genes representing each bHLH subgroup member for Arabidopsis, apple, poplar and rice (Supplementary Table S3). In apple, there were no members in groups X, XIII and XIV, indicating that gene deletions may have occurred during evolution. The numbers of apple bHLH genes within subgroups Ib(2), II, III(a + c), IVc, IVd, VII(a + b), VIIIa, IX, and XII were similar to that seen for Arabidopsis (apple to Arabidopsis ratio of between 0.7 and 1.25)^31^. Six subgroups (Ia, IVc, Va, Vb, VIIIb and XII) showed similar gene numbers between apple and poplar, while 11 subgroups (Ia, III(a + c), Iva, IVc, Vb, VII(a + b), VIIIb, VIIIc(1), VIIIc(2), XI and XII) showed similar gene number between apple and rice. The most striking difference in gene numbers within bHLH subfamilies was seen within the IIId+e subgroup, with apple exhibiting ~3-fold or greater increase in representation compared with the other three plant species. In contrast, the numbers of bHLH genes within subgroup IIIf in apple was only half or less of that seen for the other species. This suggests that these subgroups have been subject to expansion or contraction, respectively, following the divergence of apple.

### Sequence and structure analysis of apple bHLH TFs

**Figure 3:**
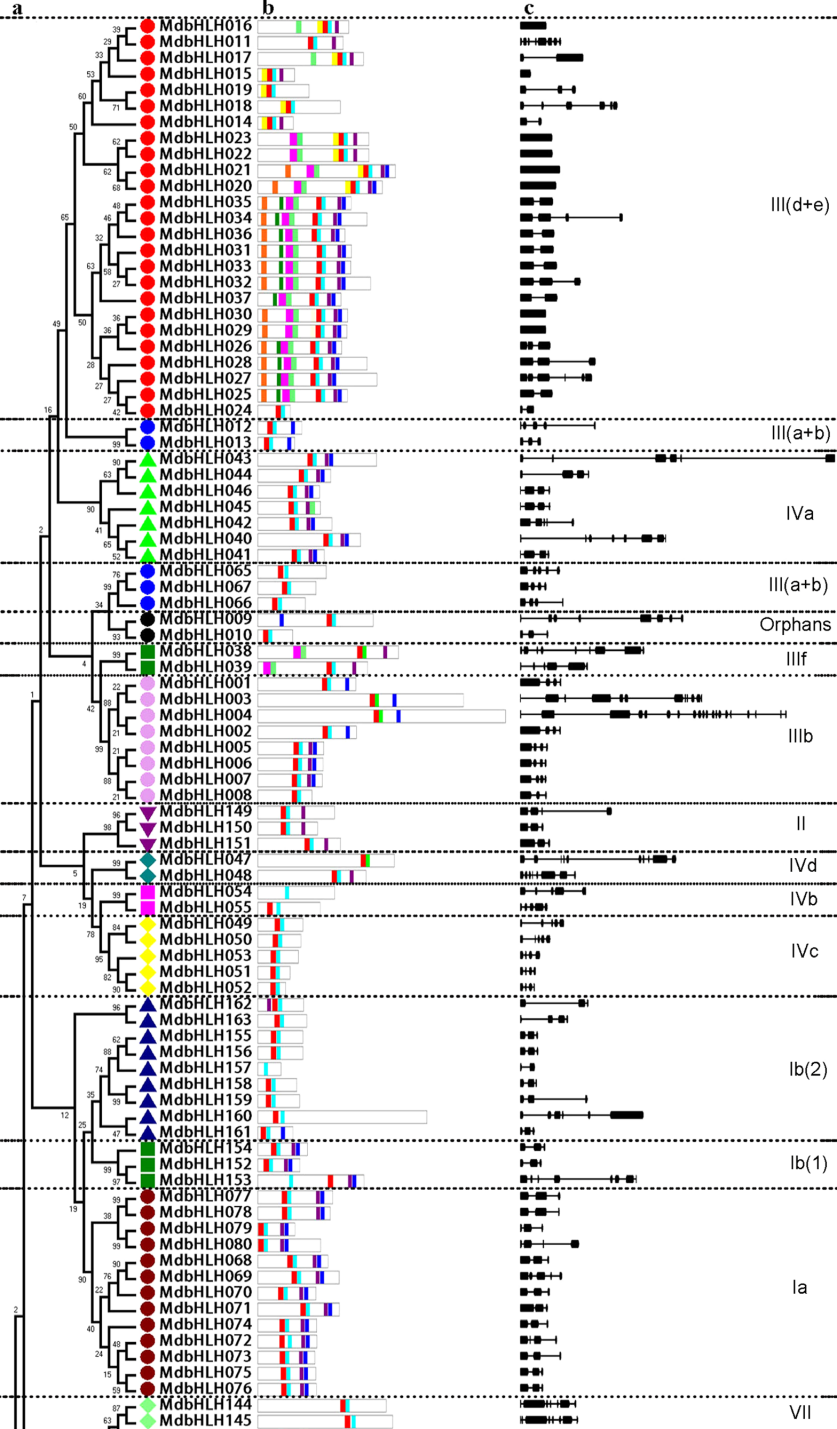

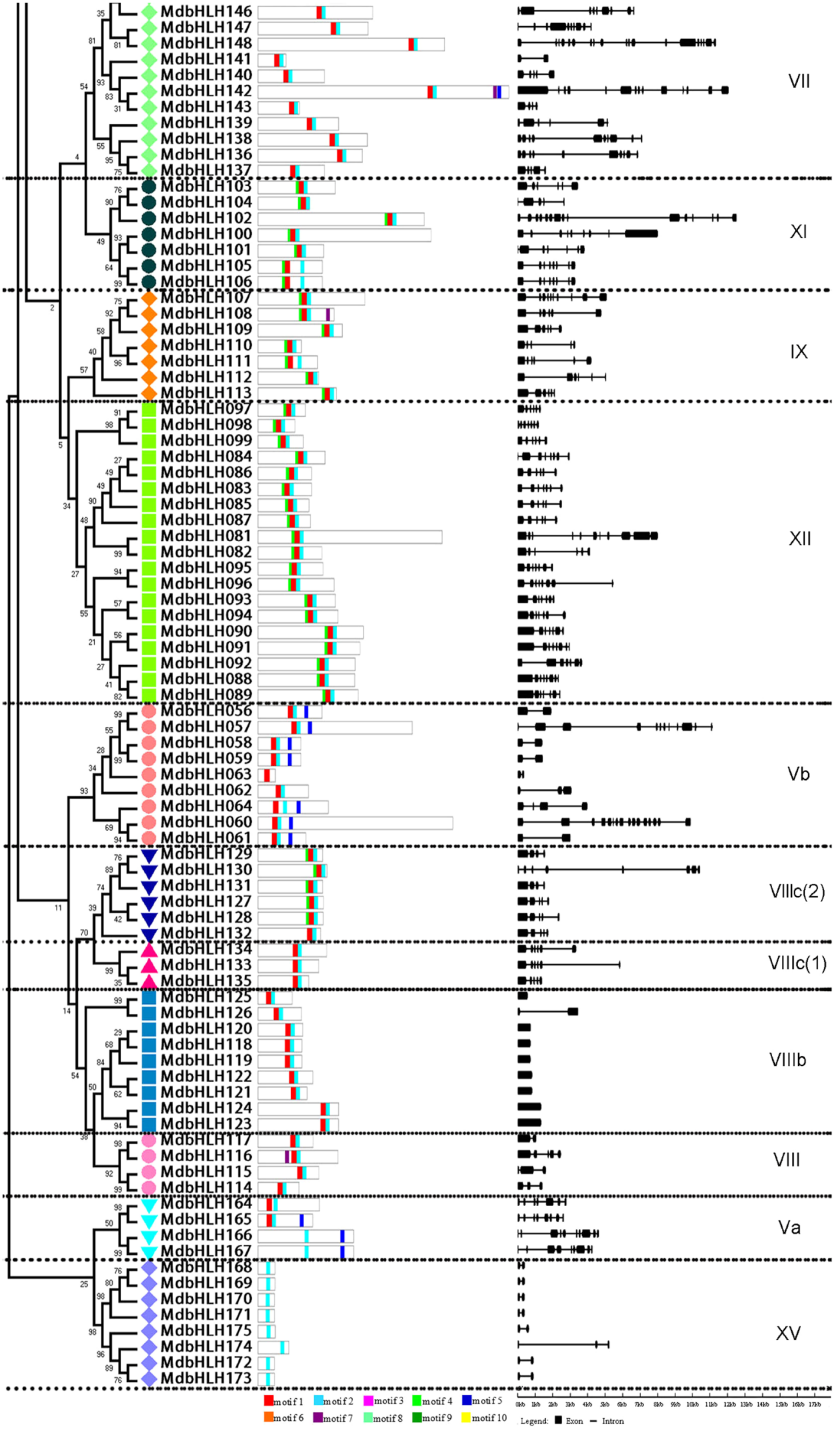
The structure of apple (*Malus*×*domestica*) bHLH transcription factors. (**a**) The phylogenetic trees were constructed using aligned domains of the MdbHLH transcription factors with MEGA 5.0 software. Reliability of the predicted tree was tested using bootstrapping with 1000 replicates. Numbers at the nodes indicate how often the group to the right appeared among bootstrap replicates. Subtrees branch lines are colored indicating different bHLH subgroups. (**b**) MEME analysis of MdbHLH protein motifs. The motifs, numbered 1–10, are depicted as different colored boxes. The sequence information for each motif is provided in Supplementary Table S4. Motifs 1 and 2 correspond to the bHLH domain. (**c**) Exon/intron structures of MdbHLH transcription factors. Exons are represented by black boxes, whereas black lines connecting two exons represent an intron. Both exons and introns were drawn to scale.

A phylogenetic tree with the 175 apple bHLH conserved domain sequences was constructed using the neighbor-joining method (Fig. 3a). The topology was consistent with that constructed from the Arabidopsis homologs (Fig. 2), and nearly all of the members in the same subgroup appeared to be clustered together. An exception was, *MdbHLH012* and *MdbHLH013*, which fell into subgroup III (a + c) in the previous analysis comparing apple and Arabidopsis (Fig. 2), but in the present study clustered with the members of subgroup IIIb. When the conserved bHLH motifs were analyzed using MEME software (http://meme-suite.org/tools/meme) (Fig. 3b and Supplementary Table S4), the members of each subgroup had similar motifs, although the lengths of the corresponding proteins were markedly different (Fig. 3b). The number of introns varied considerably among bHLH genes, ranging from zero to 19 (Fig. 3c); however, in some subgroups, the structural pattern of all members was similar. For example, none of the members of subgroup VIIIb (*MdbHLH118*-*MdbHLH124*) had an intron, while the number of introns in genes from subgroup Ia (*MdbHLH068*-*MdbHLH080*) ranged from two to four, and the corresponding proteins had a conserved motif position.

### Expansion patterns of the apple bHLH TF family

**Figure 4:**
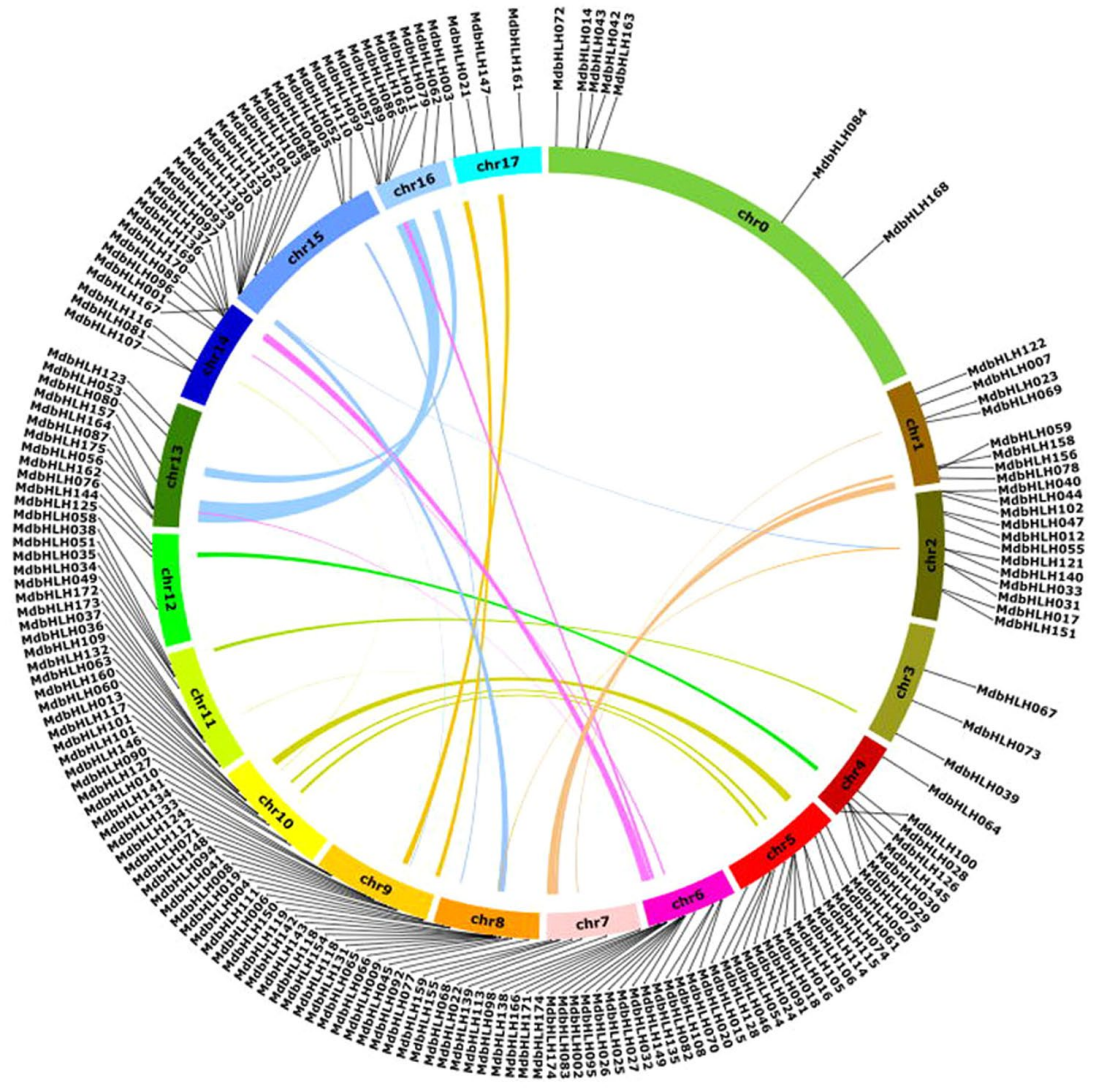
Distribution and synteny analysis of *MdbHLH* genes on the apple (*Malus*×*domestica*) chromosomes. The locations of the apple bHLH genes are indicated by vertical black lines. Colored bars connecting two chromosomal regions denote syntenic regions in apple. Chr, chromosomes.

Segmental and tandem duplications are known to be key factors driving gene family expansion^32^. In our study, the chromosomal localization of the 175 *MdbHLH* genes (Fig. 4) revealed that they were unevenly distributed among chromosomes, while 26 pairs of *MdbHLH* genes were identified that likely arose from segmental duplication (Supplementary Table S5). The analysis of tandem duplication events was based on the methods of Holub^33^, where a chromosomal region within 200 kb containing two or more genes was defined as a tandem duplication event. Accordingly, 47 genes were identified as being involved in tandem duplication events and these were associated with 23 clusters distributed among apple chromosomes 1, 2, 4, 6, 7, 8, 9, 10, 11, 14 and 16 (Supplementary Table S6).

### Synteny analysis

**Figure 5:**
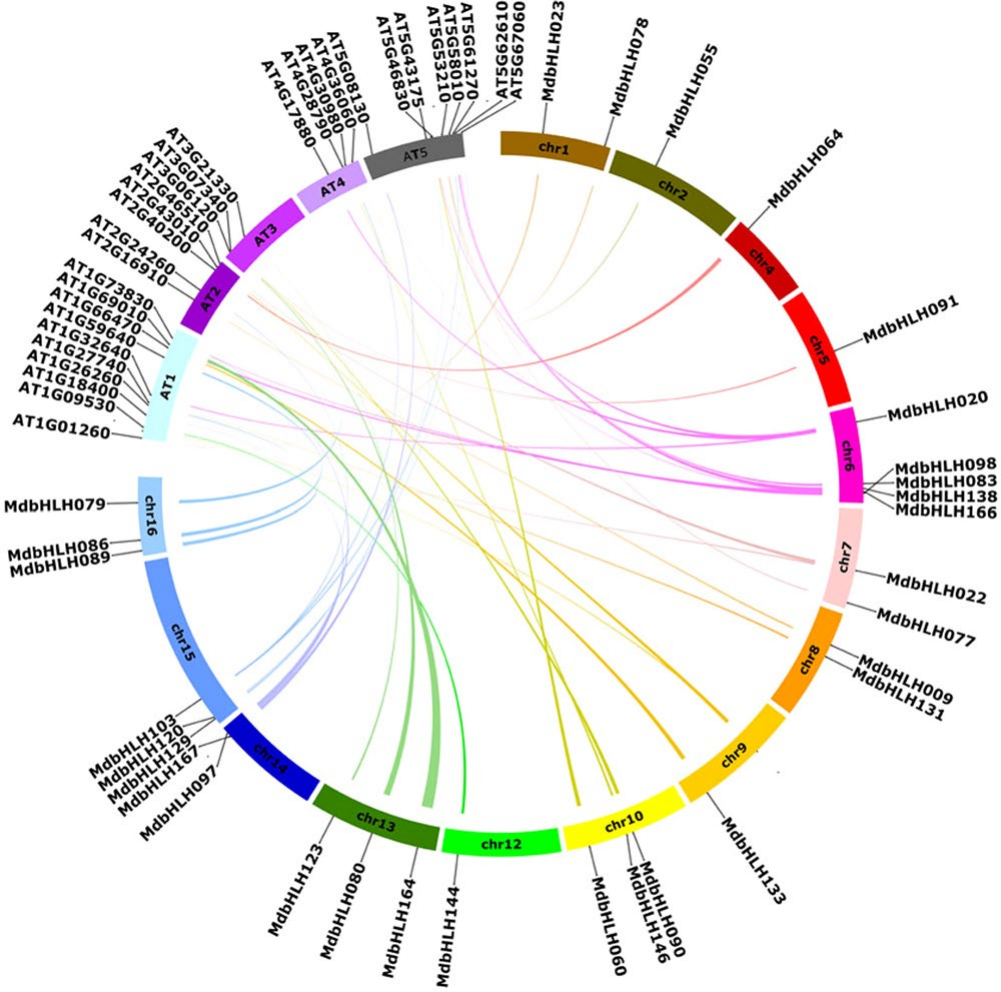
Synteny analysis of bHLH genes between apple and Arabidopsis. Apple and Arabidopsis bHLH genes are indicated by vertical black lines. Colored bars denote syntenic regions between apple and Arabidopsis bHLH genes. Chr, chromosomes.

To clarify the origin of the apple bHLH TFs and the evolutionary relationship between the apple and Arabidopsis families, a large-scale comparative synteny map was created. Forty-one pairs of bHLH genes, including 30 *AtbHLH* genes and 31 *MdbHLH* genes, showed syntenic relationships (Fig. 5, Supplementary Table S7), indicating that large-scale expansion occurred prior to the divergence of Arabidopsis and apple. Among the synteny events, many pairs were single apple to Arabidopsis correspondences, such as *MdbHLH009*-*AtbHLH021* and *MdbHLH120*-*AtbHLH088*; however, some exceptions were identified. For example, some syntenic correspondences included more than one apple gene, such as *MdbHLH077/MdbHLH078*-*AtbHLH098* and *MdbHLH090/MdbHLH091*-*AtbHLH062*, whereas others included more than one Arabidopsis gene, such as *MdbHLH020*-*AtbHLH006/AtbHLH004/AtbHLH028* and *MdbHLH022*-*AtbHLH013/AtbHLH017*. These results suggest that the many of *MdbHLH* genes share a common ancestor with *AtbHLH* genes counterparts. Despite the relatively close evolutionary relationship between the apple and Arabidopsis, the chromosomes of apple have undergone extensive rearrangement and fusions.

### Expression patterns of *MdbHLH* genes in various organs and at different developmental stages

**Figure 6:**
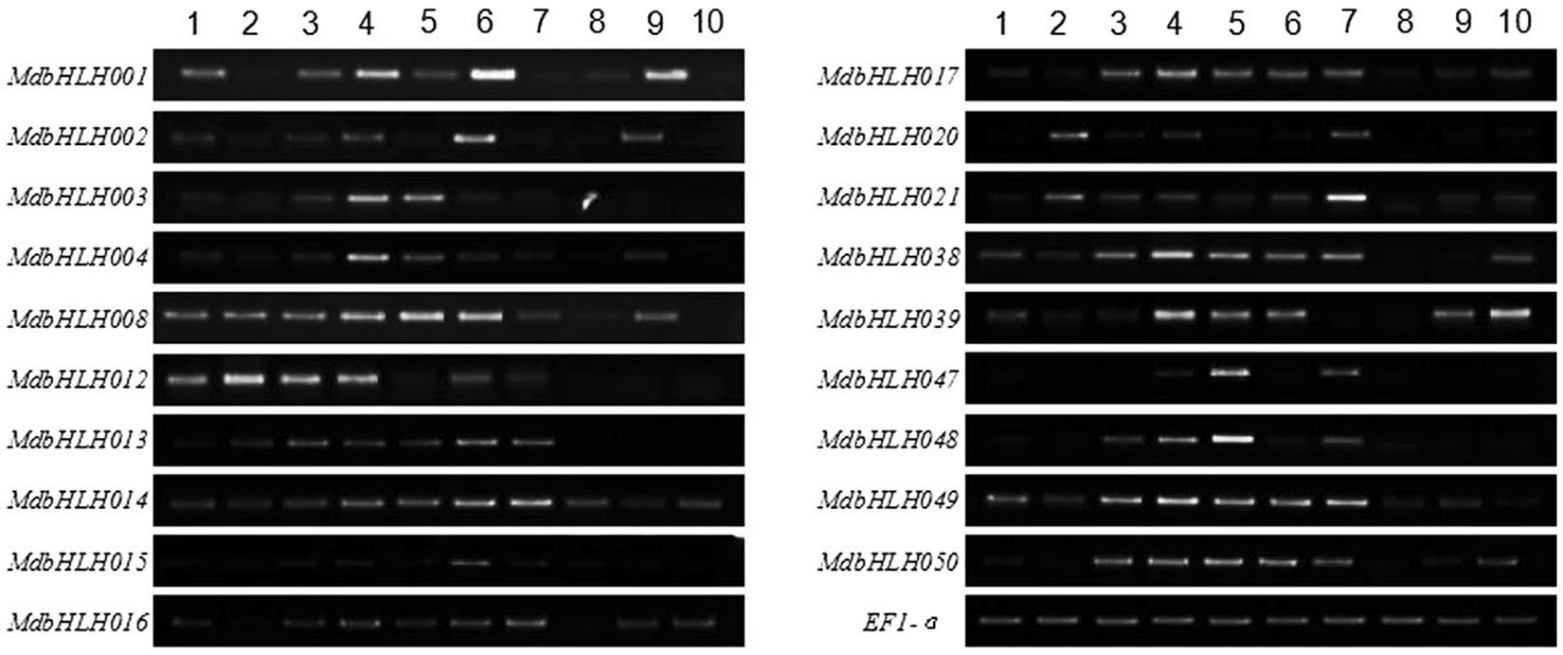
Expression profiles of 19 *MdbHLH* genes in 10 apple structures/organs. CDNA derived from the indicated structures/organs was used for the amplification of MdbHLH sequences using gene specific primers. Lanes: 1-apical buds, 2-roots, 3-stems, 4-young leaves, 5-mature leaves, 6-flower buds, 7-young fruit, 8-mature fruit, 9-seeds of young fruit, 10-seeds of mature fruit. All PCR reactions, including those amplifying the EF1-α control sequence, were carried out under similar conditions with variable number of amplification cycles. The experiments were repeated three times and the results were consistent. Representative results are shown.

Subgroup III and IV bHLH proteins may participate in plant defense and development^28,34^. Accordingly, to further verify the functions of these identified MdbHLH genes, 19 *MdbHLH* genes (*MdbHLH001*-*004*, *MdbHLH008*, *MdbHLH012*-*017*, *MdbHLH020*-*021* and *MdbHLH038*-*039* from subgroup III, and *MdbHLH047*-*050* from subgroup IV) were selected randomly to examine the expression in ten different structures and developmental stages: apical buds, roots, stems, young leaves, mature leaves, flower buds, young fruit, seeds of young fruit, mature fruit and seeds of mature fruit (Fig. 6). All 19 genes were expressed in at least one of the ten structures, although some clear spatial differences in expression were noted. For example, *MdbHLH012* was preferentially expressed in roots, *MdbHLH038* and *MdbHLH039* were highly expressed in young leaves, and *MdbHLH021* was predominantly expressed in young fruit. *MdbHLH015* and *MdbHLH002* were preferentially expressed in flower buds, and *MdbHLH047* and *MdbHLH048* were preferentially expressed in mature leaves.

### Expression of *MdbHLH* genes in response to high salt stress conditions

**Figure 7:**
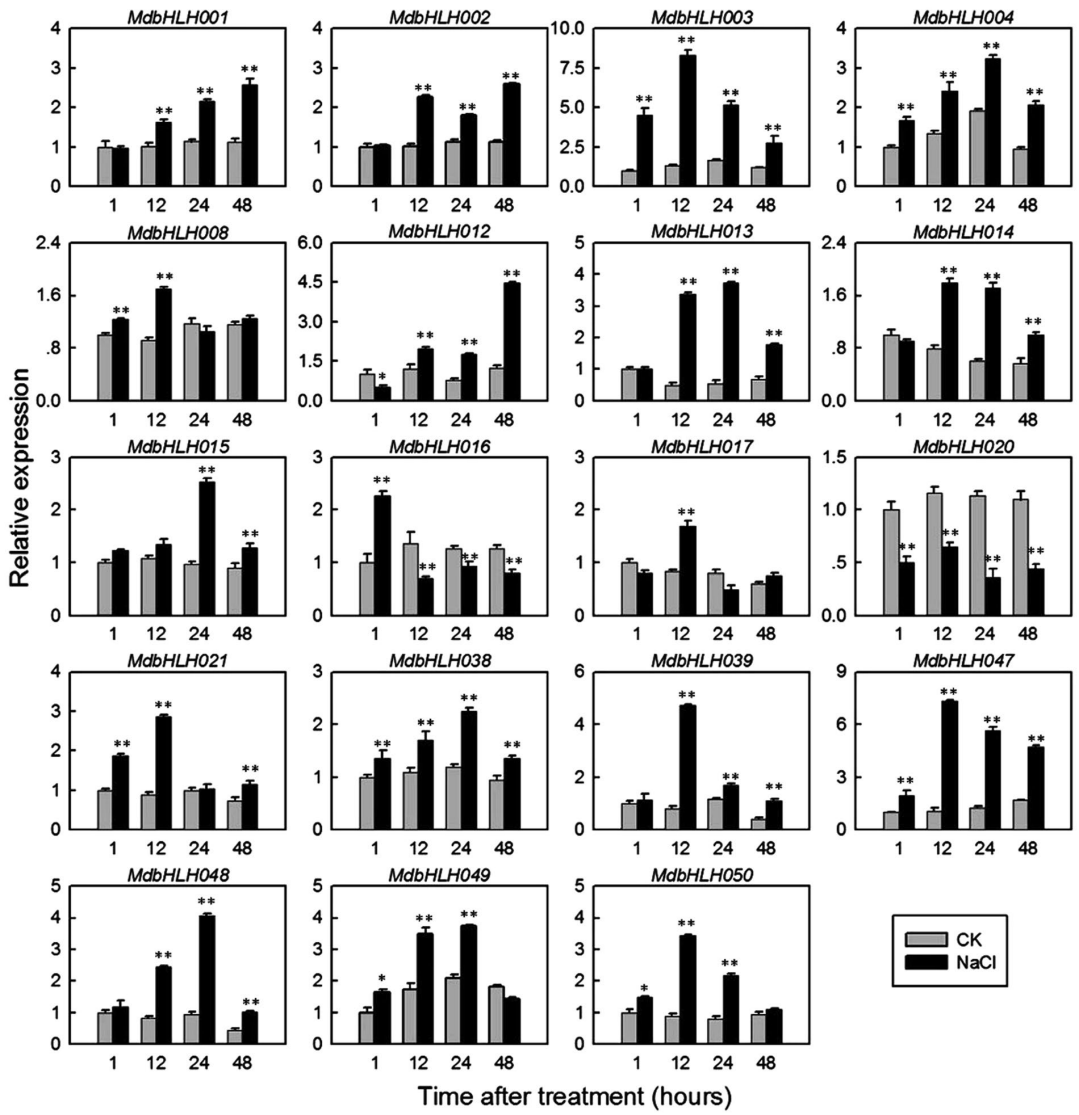
qRT-PCR analysis of 19 *MdbHLH* genes in response to salt stress. The expression levels were normalized to 1 h CK (check; deionized water) sample. Samples were harvested at 1 h, 12 h, 24 h and 48 h after 250 mM NaCl treatment or CK. Mean values and SDs were obtained from three biological and three technical replicates. Asterisks indicate the corresponding gene was significantly up- or downregulated under salt treatment (t-test; *P < 0.05, **P < 0.01).

To investigate the possible roles of the 19 selected *MdbHLH* genes in abiotic stress responses, we evaluated their expression in the leaves of apple seedlings that had been subjected to high salt stress. Almost all the genes were up-regulated, to varying degrees, except that *MdbHLH020* showed clear down-regulation (Fig. 7). However, the timing of the changes in expression varied considerably: *MdbHLH016* showed a peak of expression at a relatively early time point (1 h after the onset of the high salt treatment), whereas *MdbHLH001* and *MdbHLH012* were up-regulated at a relatively late time point (48 h after the onset of high salt treatment) (Fig. 7).

### *MdbHLH* expression in response to hormone treatments

**Figure 8:**
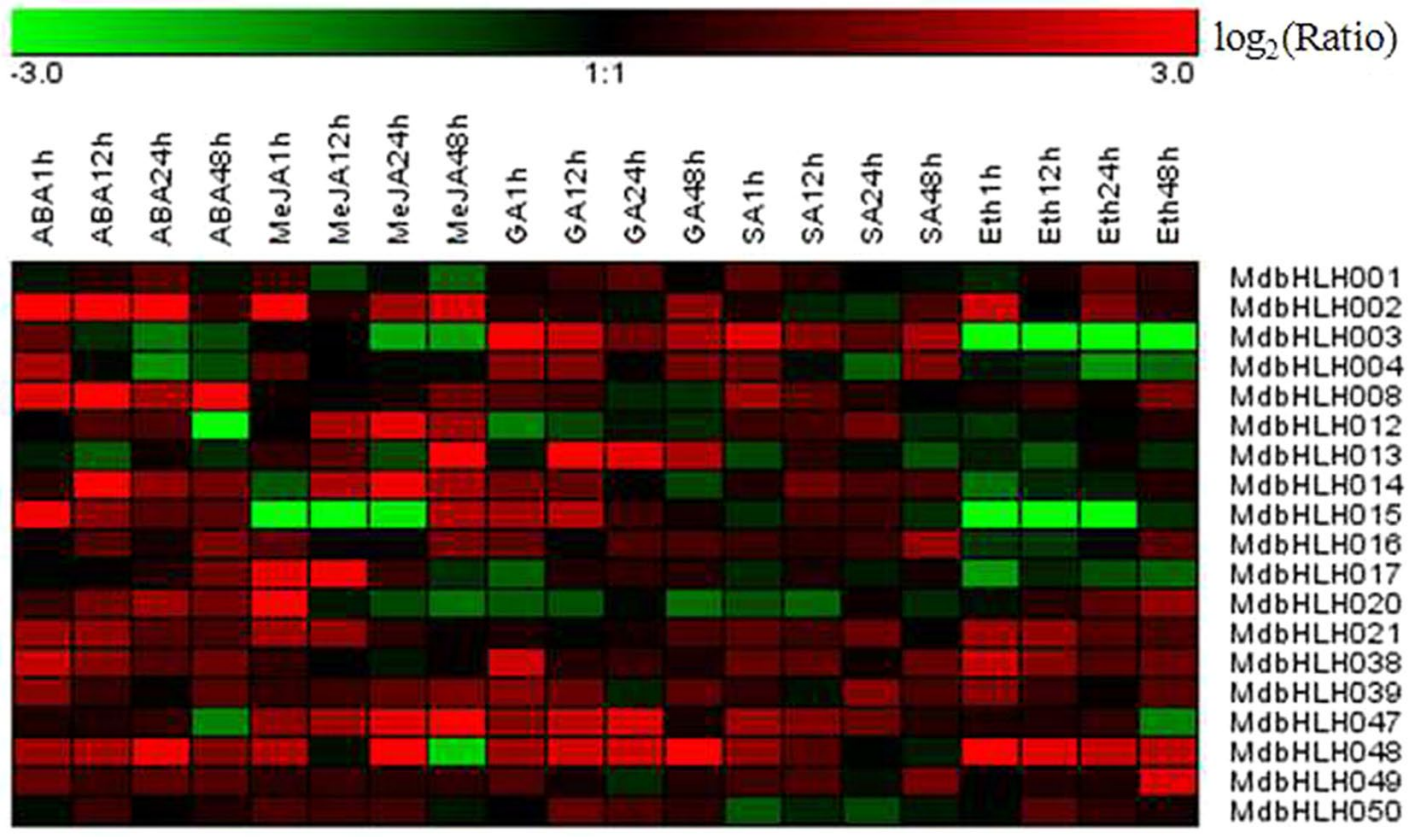
Expression profiles of 19 *MdbHLH* genes in response to various hormones. The results of the quantitative RT-PCR were analyzed using Gene Tools software, and the relative expression levels of *MdbHLH* genes under the various treatments compared to the controls were used for hierarchical cluster analysis using MeV 4.8.1. The color scale represents relative expression levels, with red indicating increased transcript abundance and green indicating decreased transcript abundance. The following five hormone treatments were tested: abscisic acid (ABA), methyl jasmonic acid (MeJA), salicylic acid (SA), gibberellin (GAs), and ethylene (Eth).

Phytohormones are critically important in coordinating regulatory networks and the signal transduction pathways associated with external cues. Abscisic acid (ABA), methyl-jasmonate (MeJA), Gibberellins (GAs), salicylic acid (SA), jasmonic acid (JA), and ethylene (Eth) have all been reported to play important roles in responses to both biotic and abiotic stresses^35^. In the present study, we examined the effects of hormone treatments on the expression profiles of selected members of Group III (*MdbHLH001*-*004*, *008*, *012*–*017*, *020*–*021 and 038*–*039*) and Group IV (*MdbHLH047*-*050*) *MdbHLHs* using real-time quantitative PCR (Fig. 8). Among the 19 targeted genes, 14 were up-regulated (defined as Log_2_-fold increase of at least 2.0 in all three biological replicates) by the ABA treatment, and the transcript levels of two genes (*MdbHLH003* and *MdbHLH004*) were highest at the first sampling time and decreased thereafter. Moreover, the transcript level of one gene (*MdbHLH013*) was high at the third sampling time but low at the other sampling times, suggesting the transcript is less stable for this gene. The other genes showed no changes in transcript levels. After MeJA treatment, 13 genes were up-regulated, including *MdbHLH012*, *MdbHLH013*, *MdbHLH047* and *MdbHLH048*, while *MdbHLH003* was down-regulated at later time points. The expression profiles after the SA, GA and Eth treatments were distinct from those modulated by ABA and MeJA, with substantial numbers of down-regulated genes being observed. After SA treatment, one gene (*MdbHLH050*) was down-regulated, and 15 were up-regulated; after GA treatment, two genes (*MdbHLH012* and *MdbHLH020*) were down-regulated, and 14 were up-regulated; after Eth treatment, four genes (*MdbHLH003*, *MdbHLH004*, *MdbHLH015* and *MdbHLH017*) were down-regulated, and 12 were up-regulated. These different transcriptional responses indicate that the *MdbHLH* gene family is collectively regulated by a broad set of hormonal signals.

## Discussion

Basic helix-loop-helix (bHLH) proteins comprise a large superfamily of eukaryotic TFs that play central roles in a wide range of metabolic, physiological and developmental processes^36^. Numerous bHLH TFs have been identified and well characterized in many plant species; however, relatively little is known about these proteins in tree species such as apple. In the present study, we identified the members of the apple bHLH TFs family and described their structure and evolutionary history. Apple is one of the most economically important fruit crops in the world, and there is considerable interest in improving its resistance to various stresses. Since bHLH TFs are known to regulate stress responses we also analyzed the expression patterns of *MdbHLH* genes in response to salt stress.

Of the 23 MdbHLH subgroups, three (Ib (2), IIId + e and XV) include only angiosperm protein encoding genes, while the remaining 20 include proteins from both angiosperms and lycophytes^3^. Since the last common ancestor of angiosperms and lycophytes dates to before 415 million years ago (Mya)^37^, this implies that these 20 bHLH subgroups are at least 415 million years (My) old. Interestingly, 18 of these subgroups include sequences from not only vascular plants but also bryophytes^3^. The oldest physical evidence for the existence of vascular land plants is trilete spores in Upper Ordovician sediments^38^, which suggests that these subgroups are more than 443 My old, and IVc subgroup contain a protein from green algae that suggests this subgroup may be over 1 billion years old^3,39^. This long evolutionary history is consistent with the apparent functional diversity of the genes observed in this study.

Gene duplication events play a major role in genomic rearrangements and expansions^40^ and are defined as either tandem duplications, with two or more genes located on the same chromosome without insertion, or segmental duplications, with duplicated genes present on different chromosomes^41^. In our study, 66 of the 175 (38%) apple *bHLH* genes that could be precisely located on chromosomes were associated with either tandem or segmental duplication events (Fig. 4 and Supplementary Tables S5 and S6). This is a lower percentage than that of duplication events in rice^26^ but still suggests that tandem and segmental duplications played a key role in the expansion of the apple bHLH family.

We conducted a MEME analysis to detect the conservation of motifs in MdbHLH gene family, and we found 10 highly conversed motifs based on all the 175 protein sets. Motifs 1 and 2 which are bHLH domains present in almost all the bHLH proteins, motifs 6 and 10 are unique in subgroup III(d + e), and the other motifs were conserved in each of the same subgroups. Taken together, all the non-bHLH conserved motifs in each subgroup support the phylogenetic relationship shown in Fig. 3a, suggesting that these extra domains are necessary for the function of each MdbHLH subgroup. The pattern of introns can provide important evidence to support phylogenetic relationships within a gene family^26^. Exon/intron diversification of gene family members has played an important role in the evolution of multiple gene families through three main types of mechanism: exon/intron gain/loss, exonization/pseudoexonization, and insertion/deletion^42^. A previous study of 167 rice *bHLH* genes found that the number of introns ranged from zero to four, and that all the genes could be assigned to ten different patterns based on the presence and positions of introns^25^. In our study, the intron number of the 175 *MdbHLH* genes varied from zero to 19, with more than 10 introns being present in only seven genes and zero to seven introns in 89% of the genes. We conclude that exon/intron gain/loss or divergence occurred during the evolutionary history of the apple bHLH family. Moreover, the gain/loss and divergence may reflect chromosomal rearrangement and fusion^32^ that has resulted in the functional diversity of the apple bHLH proteins.

Plant bHLH proteins have been associated with diverse metabolic, biosynthetic and regulatory pathways, especially those related to abiotic stress responses^43^. Prior to this current study, only a few *MdbHLH* genes have been functionally characterized: the few examples include *MdbHLH3*, which promotes anthocyanin accumulation and fruit coloration in response to low temperature^44^, *MdCIbHLH1* (Cold-Induced *bHLH1*), which encodes an ICE-like protein and is induced in response to cold stress^45^, and *MdbHLH104*, the overexpression of which in apple enhances tolerance of iron deficiency^46^. However, since approximately 40% of the Arabidopsis bHLH genes/proteins have been functionally characterized, phylogenetic/sequence analyses and sequence comparisons of apple bHLH proteins with their Arabidopsis orthologs provides an opportunity to predict the functions of the *MdbHLH* genes.

We found that selected genes belonging to subgroups III and IV exhibited diverse transcription profiles in all 10 organs/tissues examined, indicating distinct functions in apple development. The preferential expression of *MdbHLH021* in young fruit for example, might suggest a role for this gene in fruit development. *MdbHLH001* and *MdbHLH002* showed similar expression patterns in apical buds, stems, leaves, flower buds and young seeds, and are orthologous to *AtbHLH116* (also called *ICE1)*, suggesting redundant function in cold response.

Studies of bHLH subgroups have shown that subgroup IIId proteins negatively regulate JA-mediated plant defense and development^47^ and Arabidopsis TFs from bHLH subgroups IIIe and IIId negatively regulate JA-induced leaf senescence^34^. Furthermore, it was reported that a single amino acid substitution in AtMYC1 from Arabidopsis bHLH subgroup IIIf leads to trichome and root hair patterning defects by abolishing interaction with partner proteins^48^. These results suggest that the bHLH subgroup III TFs have a variety of biological functions.

ABA is often described as a stress hormone because of its notable roles in responses to stressful environments, as well as being associated with physiological processes such as storage, dehydration at later stages of embryogenesis, seed maturation, dormancy formation and abscission^49^. The observed ABA responsiveness of all the selected 19 *MdbHLH* genes, especially *MdbHLH002*, *MdbHLH004*, *MdbHLH008*, *MdbHLH014*, *MdbHLH015* and *MdbHLH048* (Fig. 8), suggests that these members of apple subgroups III and IV may be involved in stress responses. MeJA, a jasmonic acid derivative, mediates numerous transcriptional responses to wounding, herbivory and pathogenesis^50^, and the *MdbHLH002*, *MdbHLH012*, *MdbHLH013*, *MdbHLH017*, *MdbHLH020*, *MdbHLH021*, *MdbHLH047* and *MdbHLH048* genes were clearly up-regulated by a MeJA treatment, whereas *MdbHLH003* was down-regulated at a later time point. This indicates that the corresponding MdbHLH proteins are components of the MeJA-induced transcriptional network. GA stimulates plant cell elongation, promotes flowering, and releases seed/tuber dormancy^35^, and a previous study demonstrated that bHLH TFs participate in shoot branching and flower development^16–18^. Here we found that some *MdbHLH* genes were up-regulated by GA treatment, while others were down-regulated, providing evidence for the involvement of a subset of *MdbHLH* in GA-induced pathways. After Eth treatment, a clear down-regulation in the expression of *MdbHLH003*, *MdbHLH004*, *MdbHLH015*, and *MdbHLH017* was observed (Fig. 8). Given that Eth is an important signaling molecule in many processes, including root and root hair growth, cell fate determination, and responses to biotic and abiotic stress^49^, the results presented here indicate that the regulatory role of bHLH proteins under different stresses is complicated. Future studies will seek to clarify the underlying regulatory mechanisms and cross-talk between these signaling pathways.

## Materials and Methods

### Identification and annotation of the apple bHLH TF family

The bHLH conserved domain (Pfam PF00010; http://pfam.sanger.ac.uk/)^51^ was used to analyze a draft apple genome sequence^52^, as well as the GenBank non-redundant protein database and the apple genome sequence database on the website GDR (Genome Database for Rosaceae, https://www.rosaceae.org/), using HMMER (Hidden Markov Model, HMM) software^53^. Only proteins with e-values < 0.01 were considered for further analysis^54^. Then we checked the predicted bHLH transcription factors in the iTAK database (http://bioinfo.bti.cornell.edu/cgi-bin/itak/index.cgi) to retrieve any additional bHLH genes. We also used the coding sequences (CDS) to perform blast searches against the Phytozome database (http://phytozome.jgi.doe.gov/pz/portal.html#). Any additional bHLH genes were retrieved for further analysis. To further verify the reliability of those bHLH candidate sequences, domain structures analysis software SMART (http://smart.embl-heidelberg.de) was used to analyze the sequence integrity of the bHLH domain. Redundant sequences or sequences lacking of the bHLH domain were removed. Expressed sequences tags (ESTs) of apple EST database from NCBI were used for further validation. The Arabidopsis bHLH gene sequences were obtained from the Arabidopsis Information Resource (TAIR; https://www.arabidopsis.org/) using BLASTP with default parameters^53^.

### Multiple sequence alignment, phylogenetic analysis, and classification of apple bHLH genes

A total of 175 predicted MdbHLH proteins, with amino acids spanning the bHLH core domain, were subjected to a multiple sequence alignment using ClustalX 2.0 with the default parameters^55^. A further multiple sequence alignment including *MdbHLH* genes and those from Arabidopsis (*AtbHLH*) was performed using CLUSTALW^53^. The phylogenetic tree representing Arabidopsis and apple bHLH proteins was generated using MEGA 5.0 software and the neighbor-joining method^56^, with the following settings: mode, “p-distance”; gap setting, “Complete Deletion”; and bootstrap test replicate, “1,000”.

### Analyses of exon-intron structure and distribution of conserved motifs in *MdbHLH* genes

Exon–intron organization was determined based on alignments of coding sequences and genomic sequences (https://www.rosaceae.org/) and diagrams were created using the online Gene Structure Display Server (GSDS: http://gsds.cbi.pku.edu.ch). The lengths and sequence information of the conserved motifs of apple bHLH proteins, other than the bHLH domains, were obtained using MEME 4.11.2 (http://meme-suite.org/tools/meme) software with default settings, except that motif count was set to 10 and motif width to between 8 and 50.

### Tandem duplication and synteny analysis

Examples of tandem duplication were identified based on physical chromosomal location: homologous bHLH genes on a single chromosome, with no other intervening genes, were characterized as genes involved in tandem duplication events^57^. The specific physical location of each *MdbHLH* gene on its chromosome therefore determined whether it was regarded as a gene resulting from a tandem duplication event. The syntenic blocks used for constructing a synteny analysis map of the apple bHLH genes, as well as a comparison of apple and Arabidopsis bHLH genes, were obtained from the Plant Genome Duplication Database^58^, and the synteny diagrams were generated using Circos version 0.63 (http://circos.ca/).

### Plant material and treatments

*Malus* × *domestica* cv. Fuji growing on M.26 rootstocks planted at the College of Horticulture, Northwest A&F University, Yangling, China (34°20′N 108°24′E) was used in this research. Apple organs were obtained as follows: roots (newly growing lateral roots of 1–2 mm in diameter); stems (near the apices of newly growing shoots, 3–4 mm in diameter); apical buds; flower buds; young leaves; the third to fifth fully expanded young leaf beneath the shoot apex when shoots were 40~60 cm in length; mature leaves, defined as those on the middle to lower part of growing shoots; flowers; young green fruit (~60 days after full bloom); mature fruit (~100 days after full bloom); and seeds from young and mature fruit were collected from 9–10 year old field grown plants^59^.

For salt stress and hormone treatments, two-year old ‘Fuji’ seedlings growing in pots (30 cm × 26 cm × 22 cm) filled with a 5:1:1 mixture of forest soil: sand: organic substrate were used. Seedlings were irrigated with 2 L of 250 mM NaCl^59^, and leaves were collected at 1, 12, 24, and 48 h post-treatment. Plants irrigated with the same volume of water were used as a negative control. To confirm effectiveness of the salt treatment, we confirmed that the expression of SALT OVERLY SENSITIVE *MdSOS1* and *MdSOS2*, which are components of the SOS pathway^60^, was up-regulated (Supplementary Figure S2). Hormone treatments were performed by spraying leaves with 300 μM ABA, 50 μM MeJA, 100 μM SA, 100 µM GA, or 0.5 g/L Eth (released from ethephon), followed by sampling at 1, 12, 24, and 48 h after treatment. Leaves sprayed with deionized water and collected at the same time points were used as negative controls. Each treatment contained three replicates of 15 plants each. All plant samples were immediately frozen in liquid nitrogen and stored at −80 °C until RNA extraction^59,61^.

### Expression analysis of *MdbHLH* genes

Total RNA was extracted from the apple samples using an EZNA Plant RNA Kit (R6827-01, Omega Bio-tek, USA), and first-strand cDNA was synthesized by reverse transcription of 500 ng total RNA using PrimeScript RTase (TaKaRa Biotechnology, Dalian, China). The apple gene *EF1*-*α* (GenBank accession number DQ341381) with the primers F (5′-ATT CAA GTATGC CTG GGT GC-3′) and R (5′-CAG TCA GCC TGT GATGTT CC-3′) was used as an internal control. Gene-specific primers (Supplementary Table S8) were designed for the selected bHLH genes using Primer Premier 5.0. Semi-quantitative RT-PCR assays were carried out using the following profile: 94 °C for 2 min, 28–38 cycles at 94 °C for 30 s, 56–61 °C for 30 s and 72 °C for 30 s, with a final extension of 72 °C for 2 min. For each gene, the number of amplification cycles was adjusted such that an amplification product was easily apparent in at least one sample. In each case, 5 ul samples of the resulting semi-quantitative RT-PCR products were resolved on a 1.5% (w/v) agarose gel and visualized using ethidium bromide. For analysis of stress- and hormone-responsive expression, real-time quantitative PCR was conducted using SYBR green (TaKaRa Biotechnology) on an IQ5 real time-PCR machine (Bio-Rad, Hercules, CA, USA), with a final volume of 20 μl per reaction. Each reaction mixture contained 10.0 μl of SYBR Premix ExTaq II (TaKaRa Biotechnology), 1.0 μl of cDNA template, 0.8 μl of each primer (1.0 μM), and 7.4 μl of sterile distilled H_2_O. Each reaction was performed in triplicate. Cycling parameters were 95 °C for 30 s, 40 cycles at 95 °C for 5 s, and 60 °C for 30 s. Melting-curve analyses were performed using a program with 95 °C for 15 s and then a constant increase from 60 °C to 95 °C. Transcripts of the *Malus* elongation factor 1 alpha gene (*EF*-*1α*; DQ341381) were used to standardize the cDNA samples for different genes. Li^62^ had previously compared apple *EF*-*1α*, actin, and 18S rRNA as internal controls and found that *EF*-*1α* is more stable than the others as a reference gene under saline conditions. Three independent biological replications were performed for each experiment. The software IQ5 was used to analyze the relative expression levels using the normalized-expression method^63^, and the expression data from the quantitative RT-PCR were analyzed and visualized using the programs GeneSnap and Mev 4.8.1^53^.

## Electronic supplementary material

Supplementary Information
