# Supplementary material for: Identification and expression analysis of the apple (Malus × domestica) basic helix-loop-helix transcription factor family
Source: Sci Rep. 2017 Feb 9;7:28. doi: 10.1038/s41598-017-00040-y (PMC5428380; doi:10.1038/s41598-017-00040-y)
Supplement: Supplementary file 1 — Supplementary Information [file 41598_2017_40_MOESM1_ESM.pdf]

Identification and expression analysis of the apple (*Malus x domestica*) basic helix-loop-helix transcription factor family

Jinhua Yang[1,2], Min Gao[1,3], Li Huang[1,2], Yaqiong Wang[1,2], Steve van Nocker[4], Ran Wan[1,2], Chunlei Guo[1,2], Xiping Wang[1, 2]* and Hua Gao[1, 2]*

[1]State Key Laboratory of Crop Stress Biology in Arid Areas, College of Horticulture, Northwest A&F University, Yangling, China

[2]Key Laboratory of Horticultural Plant Biology and Germplasm Innovation in Northwest China, Ministry of Agriculture, Northwest A&F University, Yangling, China

[3]Department of Biological Sciences, University of Maryland, Baltimore County, 1000 Hilltop Circle, Baltimore, MD 21250, USA

[4]Department of Horticulture, Michigan State University, East Lansing, MI 48824, USA.

* Corresponding author: Xiping Wang and Hua Gao

College of Horticulture, Northwest A&F University,

Yangling 712100, Shaanxi, China

E-mail: wangxiping@nwsuaf.edu.cn, gaohua2378@163.com

Tel: 86-29-87082429

Fax: 86-29-87082613

E-mail addresses of all authors:

JY: 13759927085@163.com

MG: gaomin@umbc.edu

LH: huanglimakeit@163.com

YW: 18238800765@163.com

SVN: vannocke@msu.edu

RW: wanran2012@nwsuaf.edu.cn

CG: guo0208chun@163.com

Supplementary Figure S1: Alignment of the bHLH domain in apple

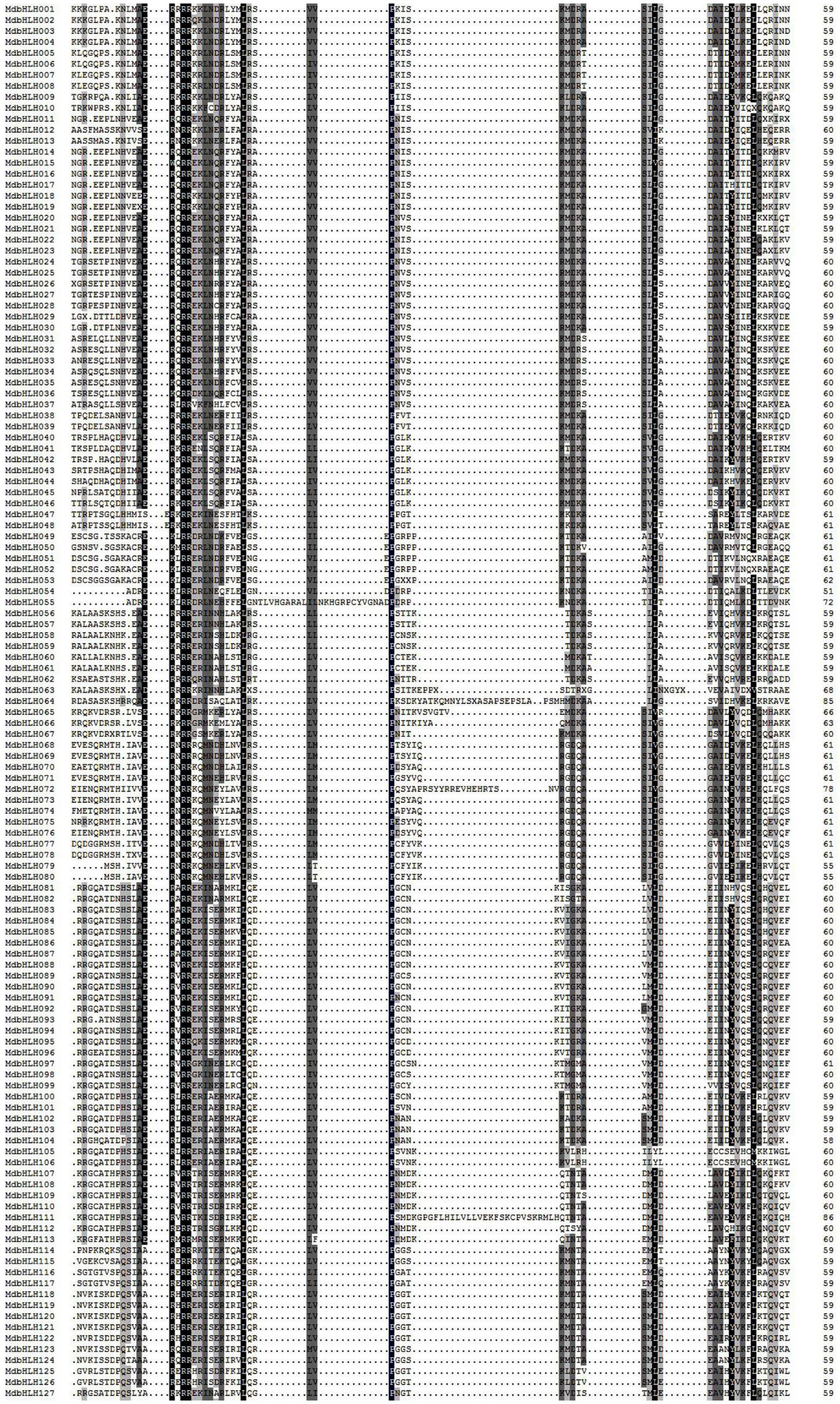

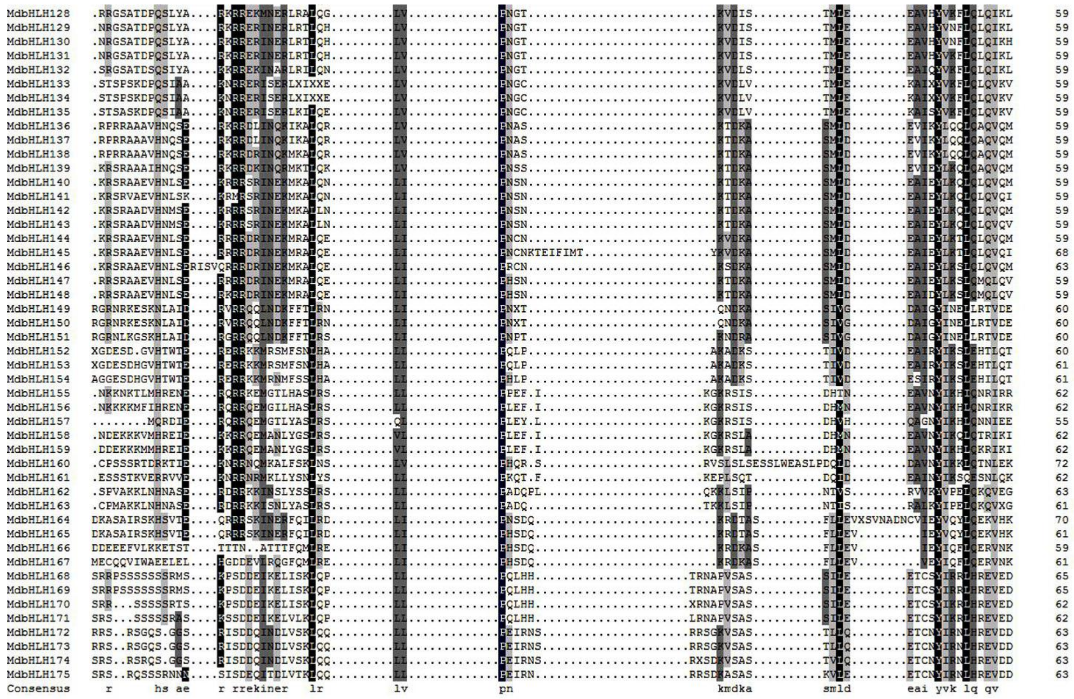

Continued

Supplementary Figure S2: Relative expression of salt-induced genes. Samples were harvested at 1 h, 12 h, 24 h and 48 h after 2L 250mM NaCl treatment or deionized water (ck).

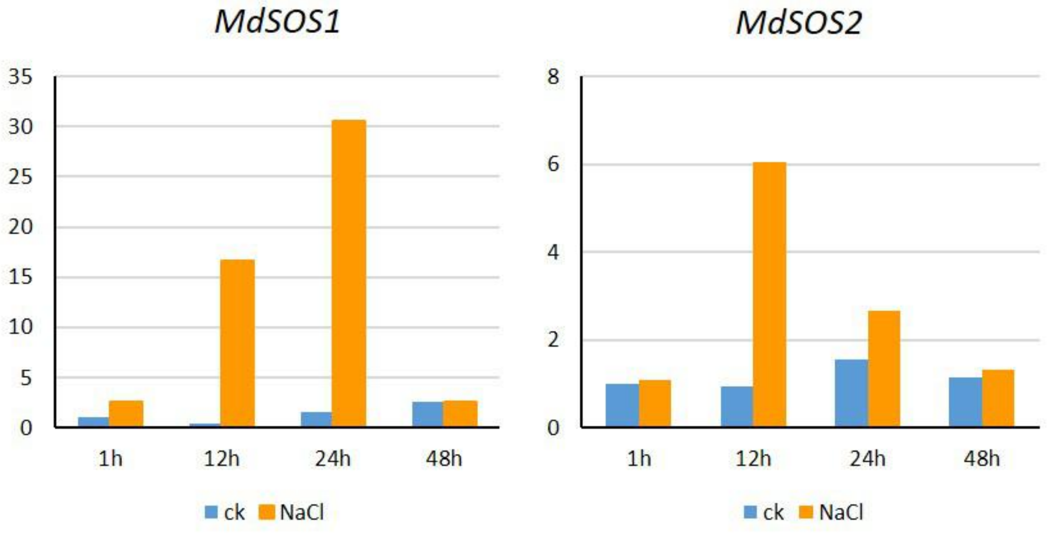

Supplementary Table S1: EST support apple bHLH genes

EST ID	length	Gene	length	query start	query end	hit start	hit end	%identity	category
CN939432.1	644	MdbHLH001	1626	377	644	1	269	99.26	overlap (query->hit)
CN900969.1	612	MdbHLH001	1626	2	612	25	635	99.84	query inclusive
CV129294.1	504	MdbHLH001	1626	1	504	677	1178	97.82	query inclusive
EG631286.1	806	MdbHLH001	1626	1	589	1038	1626	99.66	overlap (hit->query)
GO500662.1	661	MdbHLH004	4101	20	661	29	670	99.53	query inclusive
JZ481377.1	352	MdbHLH004	4101	1	344	1079	1422	99.42	query inclusive
CO756638.1	416	MdbHLH004	4101	1	407	3311	3717	97.3	query inclusive
EB155098.1	215	MdbHLH006	1086	1	215	358	572	99.53	query inclusive
CO417239.1	437	MdbHLH006	1086	9	437	555	983	98.83	query inclusive
CN895625.1	542	MdbHLH007	1071	10	542	1	534	99.44	query inclusive
GO535384.1	510	MdbHLH007	1071	19	510	113	604	99.59	query inclusive
GO538100.1	635	MdbHLH007	1071	244	635	680	1071	99.49	overlap (query->hit)
EB139931.1	661	MdbHLH008	900	45	661	1	617	98.22	overlap (query->hit)
EE663852.1	1008	MdbHLH012	735	14	748	1	735	100	hit inclusive
DT040838.1	665	MdbHLH013	615	111	665	18	572	100	overlap (query->hit)
EB139563.1	529	MdbHLH016	1509	273	529	1	257	99.22	overlap (query->hit)
EB147761.1	598	MdbHLH016	1509	1	598	552	1149	99.16	query inclusive
CO901391.1	684	MdbHLH016	1509	1	667	843	1509	98.95	query inclusive
EG631250.1	1215	MdbHLH017	1752	1	1049	704	1752	100	overlap (hit->query)
EB138570.1	646	MdbHLH020	2061	61	646	1	586	99.83	overlap (query->hit)
EG631394.1	1651	MdbHLH020	2061	1	1330	737	2061	98.28	overlap (hit->query)
EB152023.1	630	MdbHLH021	2280	1	630	146	775	99.84	query inclusive
EB129197.1	639	MdbHLH021	2280	3	639	754	1389	99.69	query inclusive
EB153957.1	644	MdbHLH021	2280	1	549	1732	2280	100	overlap (hit->query)
DT041552.1	722	MdbHLH022	1842	18	722	507	1211	100	query inclusive
GO555740.1	654	MdbHLH022	1842	341	654	1529	1842	100	overlap (query->hit)
CN876468.1	345	MdbHLH023	1842	1	345	863	1206	98.55	query inclusive
DY255348.1	603	MdbHLH023	1842	20	497	1365	1842	99.16	overlap (hit->query)
CV129910.1	418	MdbHLH026	1392	1	198	1195	1392	100	overlap (hit->query)
EB121643.1	441	MdbHLH028	1812	1	441	213	653	99.77	query inclusive
EB148216.1	559	MdbHLH029	1479	125	559	1	435	99.77	overlap (query->hit)
CO865209.1	479	MdbHLH029	1479	1	479	721	1199	99.37	query inclusive
EB138921.1	503	MdbHLH029	1479	1	503	932	1434	99.2	query inclusive
GO501601.1	667	MdbHLH030	1491	156	667	1	511	98.05	overlap (query->hit)
CO900198.1	628	MdbHLH030	1491	1	628	468	1095	99.36	query inclusive
EB128458.1	693	MdbHLH030	1491	1	654	839	1491	98.93	overlap (hit->query)
EB146963.1	610	MdbHLH037	1542	126	610	1	485	99.38	overlap (query->hit)
EB108949.1	380	MdbHLH037	1542	1	380	163	540	99.47	query inclusive
DR998460.1	647	MdbHLH037	1542	8	647	542	1181	99.53	query inclusive
GO567530.1	647	MdbHLH037	1542	150	647	1045	1542	99.6	overlap (query->hit)
EB157300.1	604	MdbHLH038	2331	309	604	1	296	100	overlap (query->hit)

EST ID	length	Gene	length	query start	query end	hit start	hit end	%identity	category
EB152940.1	679	MdbHLH038	2331	1	679	744	1422	99.71	query inclusive
EB155037.1	639	MdbHLH038	2331	1	639	1255	1893	99.22	query inclusive
EB153947.1	657	MdbHLH038	2331	1	631	1701	2331	99.84	overlap (hit->query)
CV793793.1	308	MdbHLH039	1821	1	308	1153	1460	99.68	query inclusive
GO518103.1	666	MdbHLH039	1821	18	614	1225	1821	99.5	overlap (hit->query)
EB147583.1	698	MdbHLH042	1227	183	698	1	517	99.23	overlap (query->hit)
CO069006.1	519	MdbHLH044	1212	1	463	750	1212	100	overlap (hit->query)
GO500656.1	695	MdbHLH045	1041	20	695	96	771	99.56	query inclusive
GO519159.1	646	MdbHLH049	750	8	538	220	750	99.62	overlap (hit->query)
CV466348.1	667	MdbHLH050	715	122	663	1	542	97.6	overlap (query->hit)
EE663710.1	825	MdbHLH050	715	247	823	139	715	98.79	overlap (query->hit)
CO755302.1	495	MdbHLH053	675	1	495	6	499	98.38	query inclusive
GO500165.1	688	MdbHLH053	675	25	686	14	675	98.94	hit inclusive
DR994178.1	603	MdbHLH054	1275	8	603	46	645	98.33	query inclusive
EB135645.1	640	MdbHLH054	1275	2	640	160	798	99.53	query inclusive
EB151077.1	585	MdbHLH055	1035	86	585	1	496	98.4	overlap (query->hit)
CO903071.1	603	MdbHLH055	1035	1	400	630	1035	98.03	overlap (hit->query)
CN944116.1	669	MdbHLH056	1074	437	668	1	232	99.57	overlap (query->hit)
GO518459.1	639	MdbHLH056	1074	8	136	946	1074	100	overlap (hit->query)
CO867412.1	569	MdbHLH057	2580	1	561	173	733	98.57	query inclusive
EB155597.1	638	MdbHLH059	723	54	638	1	585	100	overlap (query->hit)
CV883299.1	527	MdbHLH060	3255	274	527	1	254	100	overlap (query->hit)
GO569803.1	274	MdbHLH060	3255	1	272	365	636	98.53	query inclusive
CN946717.1	617	MdbHLH068	1164	318	617	1	300	99	overlap (query->hit)
EB141814.1	637	MdbHLH068	1164	1	578	587	1164	99.65	overlap (hit->query)
EB110102.1	434	MdbHLH070	966	19	434	1	414	98.56	query inclusive
CV657752.1	467	MdbHLH070	966	1	464	482	945	97.41	query inclusive
EG631204.1	1267	MdbHLH073	948	214	1158	1	948	99.68	hit inclusive
EG631319.1	1240	MdbHLH075	963	82	1044	1	963	100	hit inclusive
EB152090.1	726	MdbHLH076	972	27	726	1	701	99.86	overlap (query->hit)
EB119219.1	586	MdbHLH076	972	8	547	433	972	100	overlap (hit->query)
CN888172.1	618	MdbHLH081	3078	142	618	1	477	99.37	overlap (query->hit)
DT000780.1	718	MdbHLH081	3078	8	718	7	714	98.74	query inclusive
CN896307.1	655	MdbHLH082	1068	370	646	1	277	98.92	overlap (query->hit)
CN926727.1	88	MdbHLH082	1068	1	87	512	598	98.85	query inclusive
CO903484.1	618	MdbHLH082	1068	1	369	700	1068	100	overlap (hit->query)
CN491009.1	385	MdbHLH083	897	150	382	1	233	99.57	overlap (query->hit)
GO504940.1	707	MdbHLH085	855	87	707	1	625	99.2	overlap (query->hit)
GO505585.1	722	MdbHLH085	855	19	642	232	855	99.36	overlap (hit->query)
GO544435.1	649	MdbHLH086	897	38	649	1	612	99.67	overlap (query->hit)
GO548117.1	536	MdbHLH086	897	476	535	823	882	98.33	overlap (query->hit)
GO512126.1	467	MdbHLH087	879	209	467	1	259	99.23	overlap (query->hit)

EST ID	length	Gene	length	query start	query end	hit start	hit end	%identity	category
DT040570.1	580	MdbHLH087	879	18	580	7	569	100	query inclusive
GO505926.1	526	MdbHLH087	879	20	518	328	826	99.2	query inclusive
GO548117.1	536	MdbHLH087	879	460	536	804	879	98.7	overlap (query->hit)
CN907284.1	638	MdbHLH088	1620	171	638	1	468	99.79	overlap (query->hit)
CO417784.1	519	MdbHLH088	1620	1	519	544	1059	97.5	query inclusive
CN445195.1	175	MdbHLH088	1620	1	175	1291	1465	98.86	query inclusive
GO559765.1	413	MdbHLH088	1620	293	409	1504	1620	98.29	overlap (query->hit)
EE663960.1	633	MdbHLH089	1380	1	633	325	957	100	query inclusive
EB154681.1	462	MdbHLH089	1677	30	462	1	433	99.31	overlap (query->hit)
EG631384.1	1598	MdbHLH089	1677	1	1157	521	1677	99.22	overlap (hit->query)
GO518414.1	660	MdbHLH090	1764	1	660	218	877	99.39	query inclusive
EB155635.1	695	MdbHLH090	1764	1	695	677	1372	99.43	query inclusive
CN910648.1	625	MdbHLH091	1707	267	625	1	359	99.72	overlap (query->hit)
CV150834.1	419	MdbHLH091	1707	1	379	1329	1707	99.74	overlap (hit->query)
GO556908.1	334	MdbHLH092	1626	213	334	1505	1626	100	overlap (query->hit)
EG631304.1	1673	MdbHLH095	1089	192	1277	1	1089	99.45	hit inclusive
EB111505.1	384	MdbHLH096	1272	1	384	153	536	99.48	query inclusive
GO542582.1	596	MdbHLH100	2892	141	596	2437	2892	99.78	overlap (query->hit)
EB127692.1	584	MdbHLH101	1104	10	584	30	604	100	query inclusive
EB141216.1	605	MdbHLH101	1104	1	605	105	709	100	query inclusive
CN445189.1	306	MdbHLH101	1104	1	306	763	1068	97.39	query inclusive
DR997281.1	624	MdbHLH101	1104	7	316	795	1104	100	overlap (hit->query)
EB150489.1	638	MdbHLH102	2781	56	638	1	583	99.66	overlap (query->hit)
CN946612.1	543	MdbHLH102	2781	1	543	555	1096	99.08	query inclusive
CN909692.1	627	MdbHLH102	2781	1	616	837	1452	97.08	query inclusive
CO867186.1	298	MdbHLH102	2781	12	298	2463	2749	100	query inclusive
GO566953.1	643	MdbHLH102	2781	362	643	2502	2781	99.29	overlap (query->hit)
EB152771.1	690	MdbHLH103	1296	191	690	1	500	99.4	overlap (query->hit)
CO902989.1	587	MdbHLH103	1296	1	531	766	1296	99.62	overlap (hit->query)
EB152771.1	690	MdbHLH104	861	173	690	19	536	99.42	overlap (query->hit)
CO902989.1	587	MdbHLH104	861	1	60	802	861	100	overlap (hit->query)
EB146567.1	632	MdbHLH108	1272	525	632	1	108	99.07	overlap (query->hit)
GO538830.1	531	MdbHLH110	729	19	531	125	636	98.83	query inclusive
CN495896.1	480	MdbHLH111	996	157	480	1	324	98.46	overlap (query->hit)
DR996088.1	674	MdbHLH112	1020	172	669	1	498	100	overlap (query->hit)
CN861183.1	599	MdbHLH113	1314	1	408	1	408	99.75	overlap (hit->query)
DY255852.1	562	MdbHLH113	1314	7	562	117	672	98.92	query inclusive
GO567281.1	522	MdbHLH113	1314	365	522	1158	1314	98.1	overlap (query->hit)
GO521410.1	651	MdbHLH120	753	81	651	1	571	99.3	overlap (query->hit)
GO547992.1	591	MdbHLH120	753	204	591	366	753	99.74	overlap (query->hit)
CO900538.1	531	MdbHLH126	732	18	531	134	648	99.61	query inclusive
GO565624.1	506	MdbHLH126	732	364	506	590	732	100	overlap (query->hit)

EST ID	length	Gene	length	query start	query end	hit start	hit end	%identity	category
CN880856.1	546	MdbHLH130	1155	137	546	1	410	99.76	overlap (query->hit)
EB142328.1	546	MdbHLH139	1350	172	546	1	375	100	overlap (query->hit)
EB131324.1	563	MdbHLH139	1350	1	563	90	652	100	query inclusive
GO577338.1	599	MdbHLH139	1350	199	599	950	1350	99.75	overlap (query->hit)
CN884872.1	515	MdbHLH142	4194	287	515	1	229	100	overlap (query->hit)
CN544856.1	209	MdbHLH142	4194	1	207	602	808	99.03	query inclusive
CO418998.1	575	MdbHLH142	4194	1	514	3681	4194	99.03	overlap (hit->query)
EB106291.1	328	MdbHLH144	2127	223	328	1	106	100	overlap (query->hit)
CV987215.1	589	MdbHLH144	2127	1	589	462	1050	98.47	query inclusive
EB140521.1	635	MdbHLH144	2127	1	635	671	1305	99.53	query inclusive
EG631224.1	1189	MdbHLH144	2127	1	853	1275	2127	99.88	overlap (hit->query)
CN928803.1	305	MdbHLH145	2235	1	305	753	1058	98.69	query inclusive
CN945906.1	691	MdbHLH146	1920	151	691	1	535	97.78	overlap (query->hit)
CV987013.1	511	MdbHLH146	1920	1	508	84	591	99.21	query inclusive
CN948506.1	669	MdbHLH148	3123	195	669	1	475	100	overlap (query->hit)
CN874671.1	315	MdbHLH148	3123	1	313	191	501	98.4	query inclusive
CN928503.1	328	MdbHLH148	3123	1	328	663	990	99.7	query inclusive
CN930568.1	369	MdbHLH148	3123	1	369	2011	2379	100	query inclusive
CN940487.1	296	MdbHLH148	3123	1	296	2403	2698	99.66	query inclusive
DT042690.1	723	MdbHLH156	750	20	652	118	750	100	overlap (hit->query)
GO519719.1	662	MdbHLH157	387	8	268	127	387	100	overlap (hit->query)
CN932643.1	567	MdbHLH160	2802	1	567	1703	2269	99.82	query inclusive
GO528504.1	679	MdbHLH163	813	19	443	389	813	99.53	overlap (hit->query)
CO051746.1	356	MdbHLH164	1032	1	356	530	885	99.16	query inclusive
CV082136.1	679	MdbHLH165	918	326	679	1	354	99.44	overlap (query->hit)
CN444642.1	540	MdbHLH165	918	214	540	592	918	97.86	overlap (query->hit)
CN496487.1	592	MdbHLH166	1602	4	592	732	1320	100	query inclusive
GO514171.1	705	MdbHLH166	1602	20	630	994	1602	99.67	overlap (hit->query)
EB124875.1	325	MdbHLH169	288	1	225	64	288	98.22	overlap (hit->query)
EG631386.1	620	MdbHLH170	279	68	346	1	279	99.28	hit inclusive
CN492009.1	468	MdbHLH172	279	1	249	31	279	100	overlap (hit->query)
CN492009.1	468	MdbHLH173	279	1	249	31	279	100	overlap (hit->query)
CN929834.1	362	MdbHLH175	291	223	347	1	124	98.4	overlap (query->hit)

Supplementary Table S2: Information of apple bHLH transcription factors

Gene	Gene locus ID	Accession NO.	Chromosome	Start	End	ORF (aa)	protein(aa)	At ortholog locus	At locus description	score(bits)	E-value
MdbHLH001	MDP0000662999	NP_001280967.1	chr14	4133085	4135145	1626	541	AT3G26744.4	AtbHLH116, ICE1	384	e-106
MdbHLH002	MDP0000219146	XP_008374176.1	chr6	20305114	20307467	1635	544	AT3G26744.4	AtbHLH116, ICE1	382	e-106
MdbHLH003	MDP0000247912	XP_008362088.1	chr17	16539123	16541440	3405	1134	AT3G26760.1	—	357	3.00E-98
MdbHLH004	MDP0000304669	XP_008379053.1	chr9	220078	230475	4101	1366	AT5G14060.2	CARAB-AK-LYS	614	e-175
MdbHLH005	MDP0000644807	XP_008339002.1	chr15	386146	401529	1095	364	AT5G65640.1	AtbHLH093	263	1E-70
MdbHLH006	MDP0000178782	XP_008347807.1	chr8	39497642	39499213	1086	361	AT5G65640.1	AtbHLH093	269	3.00E-72
MdbHLH007	MDP0000312274	XP_008385974.1	chr1	25165210	25166707	1071	356	AT5G65640.1	AtbHLH093	285	4.00E-77
MdbHLH008	MDP0000603926	XP_008357956.1	chr9	2759788	2762319	900	299	AT5G65640.1	AtbHLH093	156	1.00E-38
MdbHLH009	MDP0000405748	XP_017189200.1	chr8	8629237	8630763	1911	636	AT2G16910.1	AtbHLH021, AMS	217	2.00E-56
MdbHLH010	MDP0000283606	XP_008365882.1	chr10	8734437	8743807	582	193	AT2G16910.1	AtbHLH021, AMS	84	4.00E-17
MdbHLH011	MDP0000790939	XP_008340043.1	chr16	7436644	7438267	1416	471	AT1G10610.1	AtbHLH090	134	1.00E-31
MdbHLH012	MDP0000318229	XP_008388700.1	chr2	4416983	4419308	735	244	AT5G57150.1	AtbHLH035	287	5.00E-78
MdbHLH013	MDP0000133262	XP_008358594.1	chr10	481873	491319	615	204	AT5G57150.2	AtbHLH035	253	6.00E-68
MdbHLH014	MDP0000279087	—	unanchored	25102619	25103818	591	196	AT4G16430.1	AtbHLH003	166	9.00E-42
MdbHLH015	MDP0000386580	XP_017180752.1	chr5	7914189	7915426	612	203	AT4G16430.1	AtbHLH003	195	1.00E-50
MdbHLH016	MDP0000401086	XP_008372342.1	chr5	29919678	29920289	1509	502	AT4G16430.1	AtbHLH003	473	e-133
MdbHLH017	MDP0000229725	NP_001287804.1	chr2	14697174	14698682	1752	583	AT4G16430.1	AtbHLH003	478	e-135
MdbHLH018	MDP0000243628	—	chr5	27625872	27629466	1368	455	AT4G16430.1	AtbHLH003	145	8.00E-35
MdbHLH019	MDP0000281689	—	chr9	14722982	14728538	852	283	AT4G16430.1	AtbHLH003	144	6.00E-35
MdbHLH020	MDP0000029168	NP_001315873.1	chr6	915551	918749	2064	687	AT1G32640.1	AtbHLH006, MYC2	593	e-170
MdbHLH021	MDP0000136498	XP_008341963.1	chr17	7090959	7093238	2280	759	AT1G32640.1	AtbHLH006, MYC2	596	e-170
MdbHLH022	MDP0000226497	XP_008376082.1	chr7	16750909	16752750	1842	613	AT1G01260.3	AtbHLH013	588	e-168
MdbHLH023	MDP0000406270	XP_008352118.1	chr1	11268083	11269924	1842	613	AT1G01260.3	AtbHLH013	598	e-171

Gene	Gene locus ID	Accession NO.	Chromosome	Start	End	ORF (aa)	protein(aa)	At ortholog locus	At locus description	score(bits)	E-value
MdbHLH024	MDP0000224302	XP_008372794.1	chr5	21098941	21099731	543	180	AT1G32640.1	AtbHLH008	110	4.00E-25
MdbHLH025	MDP0000729514	XP_008374122.1	chr6	15753085	15754931	1485	494	AT4G00870.1	AtbHLH014	203	3.00E-52
MdbHLH026	MDP0000160600	XP_008346662.1	chr6	15778994	15780715	1392	463	AT4G00870.1	AtbHLH014	194	8.00E-50
MdbHLH027	MDP0000242554	XP_008374121.1	chr6	15679753	15683857	1974	657	AT5G46760.1	AtbHLH005	165	1.00E-40
MdbHLH028	MDP0000603546	XP_008370020.1	chr4	14197949	14202248	1812	603	AT4G00870.1	AtbHLH014	199	5.00E-51
MdbHLH029	MDP0000859492	XP_008345582.1	chr4	18639107	18640585	1479	492	AT5G46830.1	AtbHLH028	189	4.00E-48
MdbHLH030	MDP0000900024	XP_008370350.1	chr4	18625181	18626671	1491	496	AT4G00870.1	AtbHLH014	225	5.00E-59
MdbHLH031	MDP0000183948	XP_008342536.1	chr2	20564654	20566569	1554	517	AT4G17880.1	AtbHLH004	187	1.00E-47
MdbHLH032	MDP0000710285	XP_008356558.1	chr6	15659005	15662450	1869	622	AT4G17880.1	AtbHLH004	194	1.00E-49
MdbHLH033	MDP0000137325	XP_008342518.1	chr2	20533312	20535408	1542	513	AT4G17880.1	AtbHLH004	182	4.00E-46
MdbHLH034	MDP0000442310	XP_008386326.1	chr11	29032700	29038535	1812	603	AT4G17880.1	AtbHLH004	204	2.00E-52
MdbHLH035	MDP0000788304	XP_017181749.1	chr11	29055691	29057560	1548	515	AT4G17880.1	AtbHLH004	224	8.00E-59
MdbHLH036	MDP0000907218	XP_008385225.1	chr11	9080188	9082133	1446	481	AT4G00870.1	AtbHLH014	189	5.00E-48
MdbHLH037	MDP0000140864	XP_008385247.1	chr11	10181535	10183650	1380	459	AT4G00870.1	AtbHLH014	157	1.00E-38
MdbHLH038	MDP0000225680	ADL36597.1	chr11	32877432	32884492	2331	776	AT4G09820.1	AtbHLH042, TT8	181	2.00E-45
MdbHLH039	MDP0000617077	XP_008368939.1	chr3	31314446	31318323	1821	606	AT4G09820.1	AtbHLH042, TT8	143	3.00E-34
MdbHLH040	MDP0000318216	—	chr2	10033711	10036472	1704	567	AT2G22760.1	AtbHLH019	183	3.00E-46
MdbHLH041	MDP0000124672	XP_008380062.1	chr9	8978359	8980018	1101	366	AT4G37850.1	AtbHLH025	175	5.00E-44
MdbHLH042	MDP0000170202	—	unanchored	10506317	10509385	1230	409	AT2G22750.2	AtbHLH018	187	1.00E-47
MdbHLH043	MDP0000291132	—	unanchored	10431696	10449680	1968	655	AT4G37850.1	AtbHLH025	233	3.00E-61
MdbHLH044	MDP0000502440	XP_008383369.1	chr2	553356	557301	1212	403	AT4G37850.1	AtbHLH025	228	4.00E-60
MdbHLH045	MDP0000159571	—	chr8	7643174	7644888	1041	346	AT4G37850.1	AtbHLH025	201	4.00E-52
MdbHLH046	MDP0000174149	XP_008372895.1	chr5	872668	874207	1026	341	AT4G37850.1	AtbHLH025	95	4.00E-50
MdbHLH047	MDP0000252899	XP_008344123.1	chr2	6225861	6235482	2262	753	AT5G56960.1	AtbHLH041	182	6.00E-46

Gene	Gene locus ID	Accession NO.	Chromosome	Start	End	ORF (aa)	protein(aa)	At ortholog locus	At locus description	score(bits)	E-value
MdbHLH048	MDP0000782908	XP_008337776.1	chr15	14949161	14952323	1794	597	AT5G56960.1	AtbHLH041	198	1.00E-50
MdbHLH049	MDP0000264803	ADL36593.1	chr11	25630582	25633098	750	249	AT5G54680.1	AtbHLH105, ILR3	250	6.00E-67
MdbHLH050	MDP0000323291	XP_008371203.1	chr5	12265466	12267873	720	239	AT5G54680.1	AtbHLH105, ILR3	267	6.00E-72
MdbHLH051	MDP0000821314	XP_008350013.1	chr11	30194158	30195060	537	178	AT3G23210.1	AtbHLH034	127	5.00E-30
MdbHLH052	MDP0000794858	—	chr15	39979052	39979882	465	154	AT3G23210.1	AtbHLH034	125	1.00E-29
MdbHLH053	MDP0000825749	XP_008391189.1	chr13	25589857	25591003	675	224	AT4G14410.1	AtbHLH104	248	2.00E-66
MdbHLH054	MDP0000275844	—	chr5	22225614	22229383	1275	424	AT3G19860.2	AtbHLH121	251	8.00E-67
MdbHLH055	MDP0000494181	XP_008336910.1	chr2	11275000	11276560	1035	344	AT3G19860.1	AtbHLH121	239	1.00E-63
MdbHLH056	MDP0000271420	XP_008389318.1	chr13	2880705	2882642	1074	357	AT1G68810.1	AtbHLH030	266	2.00E-71
MdbHLH057	MDP0000319726	XP_008339706.1	chr16	26344135	26351096	2580	859	AT1G68820.1	AtbHLH049	509	e-144
MdbHLH058	MDP0000138102	XP_008387438.1	chr12	10930018	10931426	723	240	AT2G41130.1	AtbHLH106	241	4.00E-64
MdbHLH059	MDP0000213892	NP_001281284.1	chr1	25720496	25721932	723	240	AT2G41130.1	AtbHLH106	242	2.00E-64
MdbHLH060	MDP0000249969	XP_008383635.1	chr10	27522001	27532030	3255	1084	AT5G45900.1	APG7	875	0
MdbHLH061	MDP0000256492	XP_008371455.1	chr5	5156882	5159918	801	266	AT1G68810.1	AtbHLH030	120	8.00E-28
MdbHLH062	MDP0000562627	XP_008362031.1	chr16	17842136	17845236	846	281	AT2G41130.1	AtbHLH106	78	8.00E-15
MdbHLH063	MDP0000565402	—	chr2	16373004	16373828	825	274	AT1G68810.1	AtbHLH030	88	1.00E-18
MdbHLH064	MDP0000628479	XP_008369274.1	chr4	2513528	2517527	1176	391	AT2G40200.1	AtbHLH051	115	4.00E-26
MdbHLH065	MDP0000318951	XP_008377907.1	chr8	17898094	17901442	1137	378	AT2G28160.1	AtbHLH029, FRU	214	8.00E-56
MdbHLH066	MDP0000421809	XP_008347583.1	chr8	10764111	10766590	789	262	AT2G28160.1	AtbHLH029, FRU	193	1.00E-49
MdbHLH067	MDP0000281613	XP_008360009.1	chr3	11929324	11930822	966	321	AT2G28160.1	AtbHLH029, FRU	244	7.00E-65
MdbHLH068	MDP0000829998	XP_008376237.1	chr7	19686282	19687933	1164	387	AT2G46810.1	AtbHLH070	225	4.00E-59
MdbHLH069	MDP0000321049	XP_008358042.1	chr1	13059989	13072351	1350	449	AT2G46810.1	AtbHLH070	233	3.00E-61
MdbHLH070	MDP0000794913	XP_008373582.1	chr6	4462404	4464080	966	321	AT5G46690.1	AtbHLH071	161	7.00E-40
MdbHLH071	MDP0000646823	XP_008380575.1	chr9	15744088	15745658	1353	450	AT3G24140.1	AtbHLH097, FMA	362	e-100

Gene	Gene locus ID	Accession NO.	Chromosome	Start	End	ORF (aa)	protein(aa)	At ortholog locus	At locus description	score(bits)	E-value
MdbHLH072	MDP0000160256	ADL36599.1	unanchored	2159673	2161770	981	326	AT1G72210.1	AtbHLH096	250	9.00E-67
MdbHLH073	MDP0000944210	XP_008363563.1	chr3	21015976	21018316	948	315	AT1G72210.1	AtbHLH096	268	2.00E-72
MdbHLH074	MDP0000229007	—	chr5	6389081	6390686	978	325	AT1G72210.1	AtbHLH096	199	3.00E-51
MdbHLH075	MDP0000267178	NP_001281057.1	chr4	20201361	20202671	963	320	AT1G72210.1	AtbHLH096	241	4.00E-64
MdbHLH076	MDP0000672131	XP_008388591.1	chr12	28921509	28922824	972	323	AT1G72210.1	AtbHLH096	215	4.00E-56
MdbHLH077	MDP0000131133	XP_008376691.1	chr7	24831193	24833484	1245	414	AT5G53210.1	AtbHLH098 SPCH	249	2.00E-66
MdbHLH078	MDP0000191607	XP_008354251.1	chr1	27700015	27702267	1200	399	AT5G53210.1	AtbHLH098 SPCH	161	7.00E-40
MdbHLH079	MDP0000205030	XP_008340918.1	chr16	14201562	14202872	618	205	AT3G06120.1	AtbHLH045, MUTE	228	3.00E-60
MdbHLH080	MDP0000318772	XP_008390836.1	chr13	6831541	6835839	1044	347	AT3G06120.1	AtbHLH045, MUTE	224	5.00E-59
MdbHLH081	MDP0000183819	XP_008392119.1	chr14	7715488	7723583	3078	1025	AT3G13770.1	—	915	0
MdbHLH082	MDP0000248312	—	chr6	6869339	6873746	1068	355	AT3G57800.2	AtbHLH060	285	2.00E-77
MdbHLH083	MDP0000215269	XP_017180878.1	chr6	20411821	20414407	897	298	AT5G62610.1	AtbHLH079	226	2.00E-59
MdbHLH084	MDP0000205807	XP_008374503.1	unanchored	68940825	68943829	1125	374	AT5G62610.1	AtbHLH079	237	8.00E-63
MdbHLH085	MDP0000299846	XP_008393087.1	chr14	25216848	25219388	855	284	AT5G62610.1	AtbHLH079	236	2.00E-62
MdbHLH086	MDP0000282600	XP_008339960.1	chr16	3680746	3682999	897	298	AT1G59640.1	AtbHLH031, BPE	266	1.00E-71
MdbHLH087	MDP0000195579	XP_008389558.1	chr13	5313827	5316089	879	292	AT1G59640.1	AtbHLH031, BPE	274	5.00E-74
MdbHLH088	MDP0000124019	XP_008337491.1	chr15	12542037	12544395	1620	539	AT5G48560.1	AtbHLH078	221	1.00E-57
MdbHLH089	MDP0000154272	XP_008339739.1	chr16	1881558	1884164	1677	558	AT1G68920.3	AtbHLH049	343	1.00E-94
MdbHLH090	MDP0000205113	XP_008382580.1	chr10	15347736	15350523	1764	587	AT3G07340.1	AtbHLH062	313	3.00E-85
MdbHLH091	MDP0000837139	XP_008372543.1	chr5	17680079	17683070	1707	568	AT3G07340.1	AtbHLH062	336	2.00E-92
MdbHLH092	MDP0000200355	XP_008377294.1	chr8	3042465	3046153	1626	541	AT4G34530.1	AtbHLH063, CIB1	215	6.00E-56
MdbHLH093	MDP0000231650	XP_008393688.1	chr15	1227812	1230333	1299	432	AT1G10120.1	AtbHLH074	256	3.00E-68
MdbHLH094	MDP0000313643	XP_008357997.1	chr9	7341805	7343298	1341	446	AT1G10120.1	AtbHLH074	236	2.00E-62
MdbHLH095	MDP0000225159	XP_008374189.1	chr6	16614779	16616804	1089	362	AT5G50915.2	AtbHLH137	176	3.00E-44

Gene	Gene locus ID	Accession NO.	Chromosome	Start	End	ORF (aa)	protein(aa)	At ortholog locus	At locus description	score(bits)	E-value
MdbHLH096	MDP0000410728	XP_008392769.1	chr14	21162878	21168365	1272	423	AT5G50915.2	AtbHLH137	169	3.00E-42
MdbHLH097	MDP0000779358	XP_008393318.1	chr14	27734955	27736282	795	264	AT1G73830.1	AtbHLH044, BEE3	183	1.00E-46
MdbHLH098	MDP0000879337	XP_017183603.1	chr6	23043342	23044538	621	206	AT1G73830.1	AtbHLH044, BEE3	179	1.00E-45
MdbHLH099	MDP0000725991	XP_008339657.1	chr16	1084386	1086076	759	252	AT1G25330.1	AtbHLH075	157	8.00E-39
MdbHLH100	MDP0000295593	XP_017187234.1	chr4	21390343	21398464	2892	963	AT3G04750.1	—	729	0
MdbHLH101	MDP0000176749	XP_008382959.1	chr10	22477718	22479420	1104	367	AT4G02590.2	AtbHLH059, UNE12	301	5.00E-82
MdbHLH102	MDP0000606021	XP_008384496.1	chr2	1609957	1622689	2781	926	AT2G24270.3	ALDH11A3	922	0
MdbHLH103	MDP0000534784	XP_008367760.1	chr15	8299042	8302533	1296	431	AT2G24260.1	AtbHLH066, LRL1	206	3.00E-53
MdbHLH104	MDP0000121522	XP_017184911.1	chr15	8285017	8287725	864	287	AT4G30980.1	AtbHLH069, LRL2	105	4.00E-23
MdbHLH105	MDP0000171866	XP_008372300.1	chr5	14247032	14250349	1077	358	AT4G02590.2	AtbHLH059, UNE12	197	7.00E-51
MdbHLH106	MDP0000183700	XP_008372301.1	chr5	14215258	14218575	1077	358	AT4G02590.2	AtbHLH059, UNE12	197	8.00E-51
MdbHLH107	MDP0000180376	XP_008392106.1	chr14	7619792	7624947	1791	596	AT3G12160.1	ATRABA4D	398	e-111
MdbHLH108	MDP0000423329	XP_008373739.1	chr6	6746919	6751750	1272	423	AT2G42280.1	AtbHLH130	240	1.00E-63
MdbHLH109	MDP0000307685	XP_008384525.1	chr11	28185058	28187012	1413	470	AT2G42280.1	AtbHLH130	207	2.00E-53
MdbHLH110	MDP0000269890	XP_008361617.1	chr15	42110999	42114316	729	242	AT1G35460.1	AtbHLH080	128	3.00E-30
MdbHLH111	MDP0000274003	—	chr8	26835159	26839390	996	331	AT1G35460.1	AtbHLH080	140	1.00E-33
MdbHLH112	MDP0000273574	XP_008380690.1	chr9	16610847	16615895	1020	339	AT1G05805.1	AtbHLH128	281	3.00E-76
MdbHLH113	MDP0000214472	XP_008356731.1	chr7	609432	611567	1314	437	AT2G42280.1	AtbHLH130	156	3.00E-38
MdbHLH114	MDP0000209483	XP_008372159.1	chr5	10587980	10589428	690	229	AT1G30670.1	AtbHLH052	86	1.00E-17
MdbHLH115	MDP0000280921	XP_008364271.1	chr5	10583902	10585536	1023	340	AT1G30670.1	AtbHLH052	91	2.00E-18
MdbHLH116	MDP0000216053	XP_008392315.1	chr14	10906150	10908620	1341	446	AT2G34820.1	AtbHLH053	94	2.00E-19
MdbHLH117	MDP0000791364	XP_008365998.1	chr10	24887541	24888610	927	308	AT2G34820.1	AtbHLH053	102	4.00E-22
MdbHLH118	MDP0000123893	XP_008378184.1	chr8	13184943	13185680	738	245	AT3G50330.1	AtbHLH037 HEC2	140	6.00E-34
MdbHLH119	MDP0000123894	—	chr15	12542037	12544395	738	245	AT3G50330.1	AtbHLH037 HEC2	140	6.00E-34

Gene	Gene locus ID	Accession NO.	Chromosome	Start	End	ORF (aa)	protein(aa)	At ortholog locus	At locus description	score(bits)	E-value
MdbHLH120	MDP0000249405	XP_008393796.1	chr15	2402089	2402841	753	250	AT3G50330.1	AtbHLH037 HEC2	142	3.00E-34
MdbHLH121	MDP0000420192	XP_008340184.1	chr2	16373004	16373828	825	274	AT5G67060.1	AtbHLH088, HEC1	157	9.00E-39
MdbHLH122	MDP0000711774	XP_008371659.1	chr1	3878676	3879596	921	306	AT4G00120.1	AtbHLH040 IND1	154	8.00E-38
MdbHLH123	MDP0000145937	XP_008351374.1	chr13	28270493	28271839	1347	448	AT3G21330.1	AtbHLH087	238	6.00E-63
MdbHLH124	MDP0000166245	XP_008380831.1	chr9	18326650	18327999	1350	449	AT3G21330.1	AtbHLH087	246	3.00E-65
MdbHLH125	MDP0000178778	—	chr12	19759426	19763255	576	191	AT5G01310.1	AtbHLH140, APTX	117	6.00E-27
MdbHLH126	MDP0000436338	XP_008388859.1	chr4	16225060	16228544	732	243	AT5G01310.1	AtbHLH140, APTX	117	8.00E-27
MdbHLH127	MDP0000131874	—	chr10	8918757	8920550	1098	365	AT4G33880.1	AtbHLH069, LRL2	177	1.00E-44
MdbHLH128	MDP0000946515	XP_008373007.1	chr5	24626640	24629040	1092	363	AT4G33880.1	AtbHLH069, LRL2	174	7.00E-44
MdbHLH129	MDP0000198037	XP_008393740.1	chr15	1668200	1669778	1080	359	AT2G14760.1	AtbHLH084	160	1.00E-39
MdbHLH130	MDP0000573178	XP_008393743.1	chr15	1689222	1701370	1155	384	AT1G27450.1	APT1	263	2.00E-70
MdbHLH131	MDP0000145825	XP_008357373.1	chr8	12136145	12137711	1083	360	AT4G33880.1	AtbHLH069, LRL2	164	8.00E-41
MdbHLH132	MDP0000876738	XP_008384550.1	chr11	2398431	24820752	1050	349	AT2G14760.1	AtbHLH084	162	3.00E-40
MdbHLH133	MDP0000714374	XP_008381321.1	chr9	29108727	29114887	1020	339	AT5G37800.1	AtbHLH086 RSL1	144	1.00E-34
MdbHLH134	MDP0000731330	XP_008348597.1	chr9	29116294	29122482	1152	383	AT5G37800.1	AtbHLH086 RSL1	143	2.00E-34
MdbHLH135	MDP0000435722	XP_008364556.1	chr6	12349642	12351036	852	283	AT1G66470.1	AtbHLH083, RHD6	172	2.00E-43
MdbHLH136	MDP0000319248	—	chr14	10847615	10849886	1746	581	AT4G00050.1	AtbHLH016, UNE10	117	2.00E-26
MdbHLH137	MDP0000902699	XP_008393195.1	chr14	26344194	26345832	1116	371	AT4G00050.1	AtbHLH016, UNE10	120	2.00E-27
MdbHLH138	MDP0000288670	XP_008374594.1	chr6	21547747	21554895	1830	609	AT4G00050.1	AtbHLH016, UNE10	119	9.00E-27
MdbHLH139	MDP0000439540	XP_008375803.1	chr7	10087113	10092308	1350	449	AT4G00050.1	AtbHLH016, UNE10	283	1.00E-76
MdbHLH140	MDP0000215587	XP_008340258.1	chr2	16515783	16518930	1116	371	AT4G36930.1	AtbHLH024, SPT	181	6.00E-46
MdbHLH141	MDP0000138721	—	chr9	30293302	30295057	471	156	AT1G09530.2	AtbHLH008, PIF3	107	3.00E-24
MdbHLH142	MDP0000320739	XP_008378025.1	chr8	1543934	1555640	4194	1397	AT1G45180.1	—	268	2.00E-71
MdbHLH143	MDP0000523812	XP_008347614.1	chr8	13047208	13048339	693	230	AT4G36930.1	AtbHLH024, SPT	101	4.00E-22

Gene	Gene locus ID	Accession NO.	Chromosome	Start	End	ORF (aa)	protein(aa)	At ortholog locus	At locus description	score(bits)	E-value
MdbHLH144	MDP0000290263	XP_008388421.1	chr12	27035201	27038407	2127	708	AT1G09530.2	AtbHLH008, PIF3	214	2.00E-55
MdbHLH145	MDP0000205358	XP_008370333.1	chr4	18339668	18342979	2235	744	AT1G09530.2	AtbHLH008, PIF3	189	7.00E-48
MdbHLH146	MDP0000289642	XP_017190299.1	chr10	16985872	16992614	1920	639	AT2G20180.3	AtbHLH015 PIL5	220	3.00E-57
MdbHLH147	MDP0000198404	XP_008342323.1	chr17	11357450	11361687	1842	613	AT2G20180.3	AtbHLH015 PIL5	147	3.00E-35
MdbHLH148	MDP0000254650	XP_008348351.1	chr9	11107477	11118877	3123	1040	AT1G06690.1	—	556	e-158
MdbHLH149	MDP0000275840	—	chr6	15169840	15175095	1272	423	AT2G31220.1	AtbHLH010	233	2.00E-61
MdbHLH150	MDP0000168773	XP_008378661.1	chr8	24935341	24936673	993	330	AT2G31220.1	AtbHLH010	244	7.00E-65
MdbHLH151	MDP0000945492	XP_008345255.1	chr2	28166399	28168107	1371	456	AT2G31220.1	AtbHLH010	219	3.00E-57
MdbHLH152	MDP0000484847	XP_008393789.1	chr15	2297330	2298558	702	233	AT1G49770.1	AtbHLH095, RGE1	150	1.00E-36
MdbHLH153	MDP0000617871	XP_008367662.1	chr15	2272542	2279210	1761	586	AT1G49770.1	AtbHLH095, RGE1	149	5.00E-36
MdbHLH154	MDP0000713910	XP_008378029.1	chr8	13054928	13056371	825	274	AT1G49770.1	AtbHLH095, RGE1	122	2.00E-28
MdbHLH155	MDP0000125939	XP_008376489.1	chr7	22982695	22983706	750	249	AT5G51790.1	AtbHLH120	114	4.00E-26
MdbHLH156	MDP0000246258	XP_008376681.1	chr1	24754834	24755865	750	249	AT5G51790.1	AtbHLH120	142	3.00E-34
MdbHLH157	MDP0000523715	—	chr13	12597986	12598826	387	128	AT5G51790.1	AtbHLH120	108	6.00E-25
MdbHLH158	MDP0000866611	XP_008376746.1	chr1	24819798	24820752	648	215	AT5G51790.1	AtbHLH120	154	6.00E-38
MdbHLH159	MDP0000652631	XP_008376998.1	chr7	22972901	22976730	696	231	AT5G51790.1	AtbHLH120	149	1.00E-36
MdbHLH160	MDP0000287358	XP_008383726.1	chr10	28389364	28396385	2802	933	AT4G20980.4	—	824	0
MdbHLH161	MDP0000290934	XP_008353778.1	chr17	19680685	19681503	585	194	AT5G51790.1	AtbHLH120	102	2.00E-22
MdbHLH162	MDP0000259495	XP_008389282.1	chr13	2352879	2356766	762	253	AT3G56970.1	AtbHLH038, ORG2	167	9.00E-42
MdbHLH163	MDP0000641681	XP_008389283.1	unanchored	14014082	14016827	813	270	AT3G56970.1	AtbHLH038, ORG2	146	2.00E-35
MdbHLH164	MDP0000149222	XP_008389368.1	chr13	3553012	3555824	1032	343	AT1G69010.1	AtbHLH102, BIM2	290	1.00E-78
MdbHLH165	MDP0000261293	NP_001280774.1	chr16	1967365	1970040	918	305	AT1G69010.1	AtbHLH102, BIM2	281	5.00E-76
MdbHLH166	MDP0000143208	XP_008374672.1	chr6	22283453	22288113	1602	533	AT5G08130.4	AtbHLH046, BIM1	306	3.00E-83
MdbHLH167	MDP0000242865	XP_008393259.1	chr14	26942736	26947073	1602	533	AT1G69010.1	AtbHLH102, BIM2	181	8.00E-46

Gene	Gene locus ID	Accession NO.	Chromosome	Start	End	ORF (aa)	protein(aa)	At ortholog locus	At locus description	score(bits)	E-value
MdbHLH168	MDP0000204989	XP_008351836.2	unanchored	91702832	91703237	288	95	AT1G26945.1	AtbHLH163, KDR	81	2.00E-16
MdbHLH169	MDP0000281167	XP_008351836.1	chr14	25574905	25575310	288	95	AT1G26945.1	AtbHLH163, KDR	81	1.00E-16
MdbHLH170	MDP0000218785	XP_008393138.1	chr14	25560193	25560590	279	92	AT1G26945.1	AtbHLH163, KDR	64	2.00E-11
MdbHLH171	MDP0000298705	XP_008374534.1	chr6	20886001	20886397	279	92	AT1G26945.1	AtbHLH163, KDR	59	5.00E-10
MdbHLH172	MDP0000213494	XP_008385279.1	chr11	10765479	10766386	279	92	AT1G74500.1	AtbHLH135, ATBS1,	124	1.00E-29
MdbHLH173	MDP0000228273	XP_008366350.1	chr11	10761563	10762470	279	92	AT1G74500.1	AtbHLH135, ATBS1,	124	1.00E-29
MdbHLH174	MDP0000738505	XP_008374533.1	chr6	20812656	20821375	519	172	AT1G74500.1	AtbHLH135, ATBS1,	126	7.00E-30
MdbHLH175	MDP0000210979	XP_008389506.1	chr13	4798278	4798926	291	96	AT1G26945.1	AtbHLH163, KDR	117	1.00E-27

Supplementary Table S3: Number of bHLH subfamily members identified in Arabidopsis, apple poplar, and rice. A comparison of subfamily representation between apple and Arabidopsis, poplar and rice is given as ratios.

**Subfamily**	***A. thaliana***	***M. domestica***	***P. trichocarpa***	***O .sativa***	**Ratios**		
					Malus*/*Arabidopsis	***Malus*/*Poplar***	***Malus*/Rice**
Ia	10	13	11	12	1.3	1.18	1.08
Ib(1)	1	3	1	2	3	3	1.5
Ib(2)	12	9	16	2	0.75	0.56	4.5
II	4	4	3	2	1	1.33	2
III(a+c)	3	3	8	3	1	0.38	1
IIIb	4	8	3	5	2	2.67	1.6
III(d+e)	8	26	9	5	3.25	2.89	5.2
IIIf	4	2	5	7	0.5	0.4	0.29
IVa	4	7	4	6	1.75	1.75	1.17
IVb	3	2	5	4	0.67	0.4	0.5
IVc	4	5	5	4	1.25	1	1.25
IVd	2	2	3	8	1	0.67	0.25
Va	3	4	5	3	1.33	0.8	1.33
Vb	5	9	9	9	1.8	1	1
VII(a+b)	15	13	9	14	0.87	1.44	0.93
VIIIa	4	4	2	0	1	2	-
VIIIb	6	9	9	8	1.5	1	1.13
VIIIc(1)	2	3	2	3	1.5	1.5	1
VIIIc(2)	4	6	4	7	1.5	1.5	0.86
IX	6	7	10	5	1.17	0.7	1.4
X	9	0	6	14	0	0	0
XI	5	7	5	7	1.4	1.4	1
XII	17	19	24	16	1.12	0.79	1.19
XIII	3	0	6	3	0	0	0
XIV	4	0	4	3	0	0	0
XV	4	8	4	5	2	2	1.6
orphans	12	2	11	8	0.17	0.18	0.25
total	158	175	183	165	1.11	0.96	1.06

Supplementary Table S4: Conserved motifs of apple bHLH genes

No.	Best possible match	No. of MdbHLH proteins in which motif is present	MdbHLH proteins in which motif is present
1	HS[EL]AERXRRE[KR][IL][NS]ER[FLM]KAL[RQ]S[LV]VP[NG]XSKM	163	23,22,17,16,14,21,20,33,32,31,99,15,27,25,24,30,87,86,84,83,91,85,35,39,38,90,88,34,89,28,104,103,96,100,94,93,29,92,26,82,81,95,145,144,36,102,4,3,1,101,12,106,105,13,2,120,119,118,98,128,131,130,129,135,122,121,19,124,109,108,107,46,9,61,60,148,147,139,138,42,8,7,6,5,18,44,45,40,57,56,132,97,43,123,110,127,140,37,126,125,65,11,71,137,136,143,142,67,41,79,66,112,80,111,47,59,58,62,48,115,114,73,10,164,70,72,113,77,134,133,165,78,76,141,69,68,116,75,63,117,64,151,74,146,150,149,154,163,162,153,152,52,51,50,155,53,49,159,158,156,161,160,55
2	DKAS[MI]L[DG][ED]A[IV]NY[VI]KELQXQ[VI][QK]	174	104,103,102,143,142,141,140,121,146,120,119,118,139,153,145,144,138,137,136,147,9,148,122,39,111,110,38,100,89,94,93,44,22,43,91,90,42,40,123,131,128,127,21,126,125,106,105,101,88,132,92,41,86,96,95,87,130,129,76,46,45,2,1,124,85,84,83,23,20,4,3,19,18,16,166,98,97,26,15,135,134,133,109,167,150,149,75,30,25,24,14,81,37,108,107,17,13,10,28,151,66,33,67,113,165,78,77,57,56,35,34,32,12,152,27,82,29,8,7,6,5,74,73,36,31,79,80,62,72,99,115,114,65,69,68,158,71,11,159,117,116,53,64,70,171,170,154,156,112,59,58,48,55,169,168,49,52,51,47,50,54,173,172,174,60,160,61,175,155,161,162,157,163,164
3	F[GAC][SH]GG[FH]VWL[AT]GDHE[LF]Q[FS]YECER[VA]K[EL]AR[MS][HA]GIQTLVC[IV][AP]T[PSC	19	35,34,33,28,31,32,25,36,37,30,29,27,26,21,20,23,39,38,22
4	DYI[HKR]VRA[RK]RGQATD[PS]	38	92,91,90,88,87,86,85,95,81,84,83,89,94,93,82,106,105,101,102,99,108,107,100,96,104,103,97,110,111,112,109,98,113,128,127,131,130,129
5	[AL][KR][LI][MI]SAL[EK][SD]LGL[DQ][VI][LV]HA[SN][IV][ST][ST]	60	33,32,34,36,35,28,27,26,31,25,20,78,37,4,77,2,1,3,30,29,79,68,6,5,71,21,80,69,13,76,75,70,59,11,9,154,142,60,153,152,73,72,58,7,57,56,12,61,42,40,41,74,43,44,161,166,165,46,167,64
6	[TP][FLT][QL]QRLQF[IL][IVL]QNRPEWWVY[SA]IFWQASKDS	14	33,36,31,25,35,34,32,27,28,30,29,26,20,21
7	[ED][IV][ED]VKI[SV]GS[DE][AV]M[IL][RK][VI]Q[CS]PKK	57	27,25,21,20,31,26,35,28,32,33,44,43,34,42,40,46,45,29,68,30,73,41,72,71,69,22,76,154,150,142,151,153,152,74,78,79,80,77,23,75,16,15,149,70,36,6,5,39,38,17,7,37,14,116,48,108,162
8	GV[VL]EL[AG]SL[DE]VIKEDWGLV[QH]L[SV][KR]SLFGSDN	22	31,32,33,35,34,28,27,36,37,25,26,29,30,22,39,20,38,21,23,16,17,45
9	[GH]GDVTD[SA]EWFYFYTVSLTQSF	11	33,32,25,26,37,28,35,34,31,27,36
10	K[DE]ESS[AT]QI[DP]ER[KR]PRKRGRKPANGREEPLN	12	16,19,18,15,14,17,23,22,20,21,30,29

Supplementary Table S5: Synteny blocks of bHLH genes within apple genomes

ID*	region1(*apple*)			region2(*apple*)			gene in the synteny region		gene name	
	**chr**	**start**	**stop**	**chr**	**start**	**stop**	**gene1**	**gene2**	**gene1**	**gene2**
492	chr8	11247737	13402424	chr15	950583	2516030	MDP0000123893	MDP0000249405	MdbHLH118	MdbHLH120
510	chr8	12869683	13193387	chr2	16769400	16245904	MDP0000123893	MDP0000420192	MdbHLH118	MdbHLH121
460	chr7	24072169	26572489	chr1	26971982	29362240	MDP0000131133	MDP0000191607	MdbHLH077	MdbHLH078
441	chr6	21603669	23252331	chr14	26451960	27949962	MDP0000143208	MDP0000242865	MdbHLH166	MdbHLH167
160	chr13	1863276	8818217	chr16	601180	6855456	MDP0000149222	MDP0000261293	MdbHLH164	MdbHLH165
413	chr5	13693594	14429715	chr10	20213815	19601292	MDP0000171866	MDP0000176749	MdbHLH105	MdbHLH101
70	chr10	19586691	20232980	chr5	14436033	13678925	MDP0000176749	MDP0000183700	MdbHLH101	MdbHLH106
138	chr12	24709364	26212171	chr4	16136464	17512867	MDP0000178778	MDP0000436338	MdbHLH125	MdbHLH126
502	chr8	24881052	25343297	chr15	38777791	39749711	MDP0000178782	MDP0000644807	MdbHLH006	MdbHLH005
298	chr17	11026668	12735850	chr9	10900967	12694279	MDP0000198404	MDP0000254650	MdbHLH147	MdbHLH148
267	chr16	12902222	15301326	chr13	16352214	19332046	MDP0000205030	MDP0000318772	MdbHLH079	MdbHLH080
68	chr10	15020119	16039858	chr5	17972090	16995381	MDP0000205113	MDP0000837139	MdbHLH090	MdbHLH091
180	chr13	4641471	5075438	chr6	20910626	20670507	MDP0000210979	MDP0000738505	MdbHLH175	MdbHLH174
454	chr6	20020499	21137599	chr16	4162534	2974507	MDP0000215269	MDP0000282600	MdbHLH083	MdbHLH086
185	chr14	10850550	11026068	chr10	24921153	24741559	MDP0000216053	MDP0000791364	MdbHLH116	MdbHLH117
445	chr6	16389787	17002271	chr14	20204377	20608737	MDP0000219146	MDP0000662999	MdbHLH002	MdbHLH001
92	chr11	31939391	33123840	chr3	30835619	31513203	MDP0000225680	MDP0000617077	MdbHLH038	MdbHLH039
470	chr7	16750909	17136439	chr1	11269924	10956906	MDP0000226497	MDP0000406270	MdbHLH022	MdbHLH023
118	chr11	10617322	10762470	chr6	20696228	20821375	MDP0000228273	MDP0000738505	MdbHLH173	MdbHLH174
262	chr15	1162273	1406449	chr9	9966399	10252573	MDP0000231650	MDP0000313643	MdbHLH093	MdbHLH094
31	chr1	24589367	25713327	chr7	22940799	24013794	MDP0000246258	MDP0000652631	MdbHLH156	MdbHLH159
299	chr17	9926	1688611	chr9	216181	1607515	MDP0000247912	MDP0000304669	MdbHLH003	MdbHLH004

ID	region1(*apple)*			region2(*apple*)			gene in the synteny region		gene name	
	**chr**	**start**	**stop**	**chr**	**start**	**stop**	**gene1**	**gene2**	**gene1**	**gene2**
240	chr15	2152073	2433771	chr2	16769400	16245904	MDP0000249405	MDP0000420192	MdbHLH120	MdbHLH121
62	chr10	26898052	29279309	chr5	5864081	3448927	MDP0000249969	MDP0000256492	MdbHLH060	MdbHLH061
439	chr6	21304862	24104681	chr14	26076176	28609211	MDP0000288670	MDP0000902699	MdbHLH138	MdbHLH137
245	chr15	965933	2520897	chr8	11247737	13402424	MDP0000617871	MDP0000713910	MdbHLH153	MdbHLH154

* ID refers to the synteny block ID in the synteny analysis (Nucleic Acids Res, 2012. 40(7): e49.).

Supplementary Table S6: Tandem duplication events in the 175 *MdbHLH* genes

**Cluster number**	**Chromosome**	**Gene**	**Start site**	**End site**
1	chr1	MdbHLH156	24754834	24755865
		MdbHLH158	24819798	24820752
2	chr2	MdbHLH121	16373004	16373828
		MdbHLH140	16515783	16518930
3	chr2	MdbHLH017	27625872	27629466
		MdbHLH151	28166399	28168107
4	chr2	MdbHLH040	481873	491319
		MdbHLH044	553356	557301
5	chr4	MdbHLH030	18625181	18626671
		MdbHLH029	18640585	18640585
6	chr5	MdbHLH115	10583902	10585536
		MdbHLH114	10587980	10589428
7	chr5	MdbHLH016	14697174	14698682
		MdbHLH018	14722982	14728538
8	chr6	MdbHLH032	15659005	15662450
		MdbHLH027	15679753	15683857
9	chr6	MdbHLH025	15753085	15754931
		MdbHLH026	15778994	15780715
10	chr7	MdbHLH159	22972901	22976730
		MdbHLH155	22982695	22983706
11	chr8	MdbHLH154	13054928	13056371
		MdbHLH142	13059989	13072351
12	chr8	MdbHLH118	13184943	13185680
		MdbHLH119	13186464	13187201
13	chr8	MdbHLH150	24935341	24936673
		MdbHLH006	25165210	25166707
14	chr9	MdbHLH133	29108727	29114887
		MdbHLH134	29116294	29122482
15	chr10	MdbHLH117	24887541	24888610
		MdbHLH013	25102619	25103818
16	chr11	MdbHLH173	10761563	10762470
		MdbHLH172	10765479	10766386
17	chr11	MdbHLH034	29032700	29038535
		MdbHLH035	29055691	29057560
18	chr14	MdbHLH107	7619792	7624947
		MdbHLH081	7715488	7723583
19	chr14	MdbHLH169	25574905	25575310
		MdbHLH170	25560193	25560590
20	chr15	MdbHLH129	1668200	1669778
		MdbHLH130	1689222	1701370
21	chr15	MdbHLH104	8285017	8287725
		MdbHLH103	8299042	8302533
22	chr15	MdbHLH152	2272542	2279210
		MdbHLH153	2297330	2298558
		MdbHLH120	2402089	2402841
23	chr16	MdbHLH089	1881558	1884164
		MdbHLH165	1967365	1970040

Supplementary Table S7: Synteny blocks of bHLH genes between apple and *Arabidopsis*

ID*	region1(Arabidopsis)			region2(apple)			gene in the synteny region	
	chr	start	stop	chr	start	stop	gene1	gene2
181	Chr1	109032	145684	chr7	16750909	17180593	AT1G01260 (AtbHLH013)	MdbHLH022
3	Chr1	99894	143183	chr1	11337036	10956906	AT1G01260 (AtbHLH013)	MdbHLH023
46	Chr1	2967940	3092327	chr12	27775824	27001063	AT1G09530 (AtbHLH008)	MdbHLH144
90	Chr1	6319650	6388080	chr14	27938823	27159024	AT1G18400 (AtbHLH044)	MdbHLH097
106	Chr1	8871568	9099754	chr16	888578	1976656	AT1G26260 (AtbHLH076)	MdbHLH089
193	Chr1	9636594	9696412	chr8	12365912	11851495	AT1G27740 (AtbHLH054)	MdbHLH131
176	Chr1	11558881	11800988	chr6	4746723	4133085	AT1G32640 (AtbHLH006)	MdbHLH020
107	Chr1	21909175	22372159	chr16	3680746	4800997	AT1G59640 (AtbHLH031)	MdbHLH086
205	Chr1	24729629	25049424	chr9	29282521	28053806	AT1G66470 (AtbHLH083)	MdbHLH133
171	Chr1	25603842	26007263	chr6	23060163	22073983	AT1G69010 (AtbHLH102)	MdbHLH166
51	Chr1	25788608	26612284	chr13	3207629	6570499	AT1G69010 (AtbHLH102)	MdbHLH164
82	Chr1	27645658	27787708	chr14	28526142	27580305	AT1G73830 (AtbHLH044)	MdbHLH097
169	Chr1	27714948	27841939	chr6	23513906	22405999	AT1G73830 (AtbHLH044)	MdbHLH098
335	Chr2	7261830	7361202	chr8	8964224	8582398	AT2G16910 (AtbHLH021)	MdbHLH009
272	Chr2	10229385	10346829	chr15	8023630	8385620	AT2G24260 (AtbHLH066)	MdbHLH103
317	Chr2	16726484	17021315	chr4	2171536	3348982	AT2G40200 (AtbHLH051)	MdbHLH064
343	Chr2	17800482	17899374	chr9	12186321	11082798	AT2G43010 (AtbHLH009)	MdbHLH148
328	Chr2	18991171	19093315	chr7	15724973	16752750	AT2G46510 (AtbHLH017)	MdbHLH022
435	Chr3	1823099	1909804	chr16	14442105	13473959	AT3G06120 (AtbHLH045)	MdbHLH079
399	Chr3	1783587	1946924	chr13	18348673	16481549	AT3G06120 (AtbHLH045)	MdbHLH080
357	Chr3	2255710	2383207	chr10	16052178	15000271	AT3G07340 (AtbHLH062)	MdbHLH090
481	Chr3	2327818	2376744	chr5	17543789	17887197	AT3G07340 (AtbHLH062)	MdbHLH091
404	Chr3	7457433	7515937	chr13	28757724	28233903	AT3G21330 (AtbHLH087)	MdbHLH123
603	Chr4	9792523	10007491	chr6	3785351	4901506	AT4G17880 (AtbHLH004)	MdbHLH020
606	Chr4	9876238	9980038	chr6	4601087	3757655	AT4G17880 (AtbHLH004)	MdbHLH020
527	Chr4	14143984	14220265	chr10	17553233	16985872	AT4G28790 (AtbHLH023)	MdbHLH146
550	Chr4	14962493	15110289	chr15	7931864	8385620	AT4G30980 (AtbHLH069)	MdbHLH103

ID*	region1(Arabidopsis)			region2(apple)			gene in the synteny region	
	chr	start	stop	chr	start	stop	gene1	gene2
577	Chr4	17035028	17085120	chr2	11109734	11417245	AT4G36060 (AtbHLH011)	MdbHLH055
790	Chr5	2593050	2637433	chr6	22231721	22505305	AT5G08130 (AtbHLH046)	MdbHLH166
690	Chr5	2453135	2637433	chr14	26065824	27118002	AT5G08130 (AtbHLH046)	MdbHLH167
719	Chr5	17289100	17341569	chr15	1892069	1587511	AT5G43175 (AtbHLH139)	MdbHLH129
788	Chr5	18894402	19012010	chr6	3561936	4653734	AT5G46830 (AtbHLH028)	MdbHLH020
803	Chr5	21586606	21710066	chr7	24831193	25118477	AT5G53210 (AtbHLH098)	MdbHLH077
634	Chr5	21485455	21643562	chr1	27897464	27609256	AT5G53210 (AtbHLH098)	MdbHLH078
707	Chr5	23429291	23545157	chr15	7893773	8365113	AT5G58010 (AtbHLH082)	MdbHLH103
791	Chr5	24469587	24708893	chr6	22350141	21163821	AT5G61270 (AtbHLH072)	MdbHLH138
787	Chr5	25109991	25209508	chr6	20144763	20725780	AT5G62610 (AtbHLH079)	MdbHLH083
726	Chr5	26759460	26816566	chr15	2429580	2152073	AT5G67060 (AtbHLH088)	MdbHLH120

* ID refers to the synteny block ID in the synteny analysis (Nucleic Acids Res, 2012. 40(7): e49.).

Supplementary Table S8: Primer sequences used in expression analysis of the bHLH transcription factors in apple

Gene	Gene ID (Accession No.)	Forward and reverse primer sequence	
MdbHLH001	MDP0000662999	F	ACGGTGGTGTTTGGATGGAC
	NP_001280967.1	R	CGCCCTGGATGCTGTTATTC
MdbHLH002	MDP0000219146	F	CTACCCTGCCAAATCGTATC
	XP_008374176.1	R	GCTTGCTGGATGTCTAACC
MdbHLH003	MDP0000247912	F	AATGAGGCAGAACCCAGAAG
	XP_008362088.1	R	TGGTTGCAGAGGAGCAAAT
MdbHLH004	MDP0000304669	F	TGCCGTTGAGTCCCAAATAG
	XP_008379053.1	R	TCGTGCCGCATGCTTTAT
MdbHLH008	MDP0000603926	F	TGGAGCTTAGCCAACATGG
	XP_008357956.1	R	TTGGTTGTGTGGTGGAGAAT
MdbHLH012	MDP0000318229	F	CGACTGTGGAGGATCAAGAA
	XP_008388700.1	R	CCTTCCGGAGAAAGCAGTAA
MdbHLH013	MDP0000133262	F	GTCTGAGAGGAATAGGAGGAAG
	XP_008358594.1	R	TCGAAATCGGAACCCTGAT
MdbHLH014	MDP0000279087	F	GATGAGTCCTCGGCACAAAT
	-	R	ATGGCATCACCAAGAAGAGAG
MdbHLH015	MDP0000386580	F	TGAGGGGAAACCCAGAAAGA
	XP_017180752.1	R	GGTAATGGCATCACCAACAA
MdbHLH016	MDP0000401086	F	TGGGTGAATGACGAAGACAAG
	XP_008372342.1	R	CCACCAGATTTCGAGCTAACA
MdbHLH017	MDP0000229725	F	TGGAGGCAGTGCAGAATAAA
	NP_001287804.1	R	AGGACCACAGTGTGAAATCG
MdbHLH020	MDP0000029168	F	ATAAAGACGAGCGGGACAAG
	NP_001315873.1	R	CTGAGTCATGGACACCAGAAA
MdbHLH021	MDP0000136498	F	GTCAGAGGATGAACTGAAAGG
	XP_008341963.1	R	CTACAGAAACGCTGGCATAG
MdbHLH038	MDP0000225680	F	GACACACATTACTCGGAGAC
	ADL36597.1	R	GGAGGAACGGTACACTAAAC
MdbHLH039	MDP0000617077	F	CACCATACTCAGGGACAATC
	XP_008368939.1	R	GAGTTCTCGTCACGGTATTT
MdbHLH047	MDP0000252899	F	CTCATCAACAAGCCATCCAAG
	XP_008344123.1	R	TAAGTGGCGATGAGCAGTAG
MdbHLH048	MDP0000782908	F	CCGGGAACCAAGAAAGACAA
	XP_008337776.1	R	CGCGTAACCGTAAGACTACATC
MdbHLH049	MDP0000264803	F	CGGACAAGGCTGCTATTT
	ADL36593.1	R	AGTTGCTGTTCCAACTTCTC
MdbHLH050	MDP0000323291	F	CAACATCGAGCGTCAGATTA
	XP_008371203.1	R	TGGTCCTGTGAGGTATCAA
